# Engineering FcRn binding kinetics dramatically extends antibody serum half-life and enhances therapeutic potential

**DOI:** 10.1186/s13036-025-00506-y

**Published:** 2025-04-18

**Authors:** Sanghwan Ko, Migyeong Jo, Munsu Kyung, Wonju Lee, Woo Hyung Ko, Jung-Hyun Na, Youn Seo Chun, Byoung Joon Ko, Sang Taek Jung

**Affiliations:** 1 Department of Biomedical Sciences, Graduate School, Korea University, Seongbuk-gu, Seoul, 02841 Republic of Korea; 2 Institute of Chemical Processes, Seoul National University, Gwanak-gu, Seoul, 08826 Republic of Korea; 3 Department of Chemical and Biological Engineering, College of Engineering, Seoul National University, Gwanak-gu, Seoul, 08826 Republic of Korea; 4 BK21 Graduate Program, Department of Biomedical Sciences, Korea University College of Medicine, Seoul, 02841 Republic of Korea; 5 School of Biopharmaceutical and Medical Science, Sungshin Women’s University, Gangbuk-gu, Seoul, 01133 Republic of Korea; 6 Interdisciplinary Program for Bioengineering, Seoul National University, Seoul, 08826 Republic of Korea; 7 Present Address: Manufacturing Science & Technology Team, Manufacturing Science Group2, Samsung Bioepis, Incheon, Republic of Korea

**Keywords:** Therapeutic antibody, FcRn, pH-dependent FcRn binding kinetics, Endogenous IgG competition, Serum half-life, Complement-dependent cytotoxicity

## Abstract

**Background:**

Optimizing the IgG Fc domain for neonatal Fc receptor (FcRn) binding is crucial for enhancing antibody pharmacokinetics. The prolonged serum half-life of IgG antibody is governed by its pH-dependent interaction with FcRn, enabling efficient binding at acidic endosomal pH, intracellular trafficking, and release at neutral serum pH. However, a critical yet previously unrecognized challenge in Fc engineering for extending the serum half-life of therapeutic antibodies is the intense competition with endogenous IgG for FcRn binding during intracellular trafficking, which limits FcRn-mediated transport and reduces the serum persistence of therapeutic antibodies. To address this, we developed an Fc variant that precisely modulates pH-dependent FcRn binding kinetics, accelerates FcRn association at acidic pH, and promotes rapid dissociation at neutral pH, thereby enhancing FcRn-driven intracellular transport, outcompeting endogenous IgG, and achieving unprecedented improvement in the serum half-life of therapeutic antibodies.

**Results:**

Using comprehensive site-directed saturation mutagenesis coupled with functional screening, we generated a diverse panel of Fc variants and identified two with distinct FcRn binding kinetics: YML (L309Y/Q311M/M428L), which exhibited superior FcRn association at acidic pH and accelerated dissociation at neutral pH, and EML (L309E/Q311M/M428L), which displayed attenuated binding kinetics. In human FcRn transgenic mice, YML extended the serum half-life of clinically used trastuzumab with a wild-type Fc by 6.1-fold, demonstrating a remarkable improvement over previously reported Fc-engineered variants, including PFc29 (Q311R/M428L) and DHS (L309D/Q311H/N434S), which represent the most effective Fc modifications for prolonging serum persistence to date. This in vivo validation underscores the pivotal role of FcRn kinetic tuning in overcoming endogenous IgG competition and maximizing FcRn-mediated antibody transport. Additionally, YML exhibited potent complement-dependent cytotoxicity (CDC) while maintaining favorable physicochemical properties.

**Conclusion:**

This study presents a rational Fc engineering framework to optimize FcRn binding kinetics, addressing a previously unconsidered challenge—endogenous IgG competition during intracellular trafficking of therapeutic antibodies. The distinct kinetic behaviors of YML and EML highlight the critical necessity of precise control over pH-dependent association and dissociation rates in FcRn binding. YML represents a next-generation Fc platform, offering enhanced pharmacokinetics and improved effector functions, thus providing a powerful strategy for developing biologics with superior serum persistence and therapeutic efficacy.

**Supplementary Information:**

The online version contains supplementary material available at 10.1186/s13036-025-00506-y.

## Introduction

Therapeutic antibodies with extended serum half-lives provide significant advantages in treating a broad spectrum of diseases, including cancer, chronic inflammatory disorders, and infectious diseases, where sustained systemic drug availability is essential. By ensuring prolonged target engagement, these antibodies enhance therapeutic efficacy, reduce dosing frequency, and ultimately improve patient quality of life [1, 2]. As a result, substantial research efforts in academia and industry have been directed toward engineering antibodies with optimized pharmacokinetic profiles to maximize their therapeutic utility.

Among the various factors influencing IgG half-life—such as hydrophobicity [3], isoelectric point [4], and overall charge [3]—and clearance through on-target or off-target interactions [5, 6] influence antibody half-life, the primary determinant of IgG serum persistence is its interaction with the neonatal Fc receptor (FcRn) [7–9]. FcRn, a heterodimer consisting of an MHC class I-like heavy chain and a β2-microglobulin (β2m) subunit, governs the pH-dependent intracellular trafficking of IgG, a finely tuned process integral to antibody homeostasis and humoral immunity [2, 10, 11]. At acidic endosomal pH (5.5–6.5), FcRn binds IgG via protonated histidine residues (H310, H433, H435), facilitating receptor-mediated recycling and transcytosis. At physiological pH (7.4), deprotonation of these residues induces IgG release, allowing efficient re-entry into circulation while preventing lysosomal degradation [2, 11–13]. This dynamic FcRn-IgG interaction, characterized by iterative binding and dissociation cycles, dictates the prolonged serum persistence of IgG and represents a fundamental parameter in antibody engineering strategies.

Leveraging this mechanistic framework, researchers have developed Fc-engineered variants designed to extend serum half-life by enhancing pH-dependent FcRn equilibrium binding affinity [14–21]. Notable variants, including M252Y/S254T/T256E (YTE) [15] and M428L/N434S (LS) [14], exhibit increased FcRn binding at acidic pH, thereby improving receptor-mediated recycling and extending in vivo circulation. These modifications have driven major advancements in antibody-based therapeutics, with YTE incorporated into FDA-approved Beyfortus^®^ (nirsevimab-alip) for RSV prophylaxis in infants [22] and Evusheld^®^ (tixagevimab/cilgavimab) for SARS-CoV-2 prophylaxis [23], while LS has been utilized in Ultomiris^®^ (ravulizumab-cwvz) for paroxysmal nocturnal hemoglobinuria (PNH) [24], as well as in sotrovimab for SARS-CoV-2 [25]. These examples highlight the profound clinical impact of Fc engineering in refining antibody pharmacokinetics and extending serum half-life.

Despite these advancements, a persistent challenge in Fc engineering lies in achieving a finely balanced interplay between high-affinity FcRn binding at endosomal pH and efficient IgG dissociation at neutral pH. Many Fc variants that exhibit enhanced FcRn affinity at acidic pH also display excessive retention at physiological pH, impairing receptor dissociation and leading to intracellular accumulation and degradation [26–28]. This limitation ultimately constrains half-life extension, underscoring the necessity of engineering Fc variants with accelerated FcRn dissociation at neutral pH while preserving strong engagement at acidic pH. To address this, Lee et al. developed the DHS variant (L309D/Q311H/N434S), which enhances human FcRn (hFcRn) binding five-fold at pH 5.8 while maintaining minimal interaction at pH 7.4. While trastuzumab-DHS exhibits lower hFcRn affinity at acidic pH than YTE and LS, its rapid dissociation at neutral pH confers a distinct pharmacokinetic advantage, resulting in significantly prolonged half-life in hFcRn transgenic mice compared to YTE and LS. These findings underscore the critical role of precisely engineered hFcRn dissociation kinetics at physiological pH in maximizing antibody serum persistence [16].

Building upon these insights, we devised an innovative Fc engineering strategy to mitigate the kinetic constraints imposed by competitive interactions between therapeutic antibodies and endogenous IgG within the endosomal and serum environments. In endosomes, hFcRn is present at approximately 50 µM [29, 30], whereas serum IgG concentrations often exceed 100 µM [31, 32], creating a highly competitive landscape for hFcRn binding. Antibodies that fail to efficiently engage hFcRn within the transient endosomal window are rapidly degraded upon lysosomal fusion [33]. Thus, for effective recycling, therapeutic antibodies must not only demonstrate high-affinity engagement with hFcRn at acidic pH to outcompete endogenous IgG but also exhibit rapid dissociation at neutral pH to facilitate efficient re-entry into circulation.

To address these challenges, we employed a systematic site-directed saturation mutagenesis and functional screening approach to refine the previously developed PFc29 variant (Q311R/M428L) [17, 34], ultimately identifying a next-generation Fc variant with meticulously tuned hFcRn binding kinetics. In hFcRn transgenic mice, this engineered Fc variant significantly extended the serum half-life of trastuzumab by 6.1-fold, surpassing the DHS variant, which had previously outperformed YTE and LS variants. Furthermore, the optimized Fc variant retained an optimal physicochemical profile and demonstrated a substantial enhancement in complement-dependent cytotoxicity (CDC) compared to wild-type and DHS-modified antibodies. These findings emphasize the transformative potential of this Fc-engineered antibody in advancing therapeutic efficacy, reducing dosing frequency, and enhancing patient outcomes, representing a paradigm shift in antibody engineering.

## Materials and methods

### Reagents

All plasmids and primers used in this study are summarized in Supplementary Tables 1 and Supplementary Table 2. Restriction endonucleases, T4 DNA ligase, and Vent polymerase were purchased from New England Biolabs (Ipswich, MA, USA). Taq polymerase and oligonucleotide primers were sourced from Biosesang (Seongnam, Republic of Korea) and Cosmogenetech (Seoul, Republic of Korea), respectively. Difco™ Terrific Broth, Ni-NTA agarose, Protein A agarose, and glutathione agarose 4B were obtained from Becton Dickinson Diagnostic Systems (Sparks, MD, USA), Qiagen (Hilden, Germany), GenScript (Scotch Plains, NJ, USA), and Incospharm (Daejeon, Republic of Korea), respectively. The 1-Step Ultra-TMB substrate solution, SYPRO™ orange protein gel stain, GIBCO FreeStyle™ 293 expression medium, and sheep anti-hC1q-polyclonal antibody-HRP conjugate were purchased from Thermo Fisher Scientific (Waltham, MA, USA). Polyethyleneimine (PEI)-Max and C1q protein were purchased from Polysciences (Taipei, Taiwan) and Quidel (San Diego, CA, USA), respectively. Goat anti-GST-HRP conjugate and CM5 sensor chip were purchased from Cytiva (Marlborough, MA, USA). HPLC-grade UPLC solvents were obtained from Fisher Scientific (Fair Lawn, NJ, USA), ensuring high purity for analytical applications. The Octet NTA biosensor was sourced from Sartorius (Göttingen, Germany), and the FcRn affinity column Gen2 was generously provided by Roche Diagnostics Korea (Seoul, Republic of Korea). FITC Annexin V Apoptosis Detection Kit with 7-AAD and Annexin V binding buffer were purchased from BioLegend (San Diego, CA, USA). Unless otherwise stated, all other biochemical reagents were sourced from Sigma-Aldrich (St. Louis, MO, USA).

### Chromatographic analysis and mass spectrometric profiling of antibodies and antibody-Fc variants

Antibodies and antibody-Fc variants were separated by size exclusion chromatography (SEC) using a Biosuit High-Resolution SEC column (7.5 × 300 mm, 250 Å particle size). Separation was performed with isocratic flow in PBS (pH 7.4) on a Waters Alliance 2695 system, with a UV/Vis detector (2489), monitored by a UV/Vis detector at 280 nm. For intact mass analysis, a Waters ACQUITY I-Class UPLC system (Milford, MA, USA) coupled with a Thermo MabPac™ RP column (2.1 mm × 50 mm, 4 μm particle size) was used. Eluent A consisted of 0.1% formic acid in water, while eluent B comprised 0.1% formic acid in 100% acetonitrile. Separation was performed at a flow rate of 0.2 ml/min, with the gradient maintained at 20% eluent B for 2 min and then linearly increased to 50% eluent B over 8 min. Effluents were analyzed using a Waters Synapt G2-Si HDMS system. The system parameters were optimized for high-resolution data acquisition, with a capillary voltage of 3 kV, a source temperature of 150 °C, and a desolvation temperature of 80 °C. Glycan profiling of antibodies and antibody-Fc variants followed the methodology described in our previous publication [17], ensuring precise characterization of glycosylation profiles.

### Surface plasmon resonance analysis

The Biacore T200 instrument was utilized to determine the affinities of trastuzumab-Fc variants for hFcRn at pH 6.0. Trastuzumab-Fc variants were immobilized on a Series S CM5 sensor chip using amine coupling chemistry to achieve approximately 1,000 RU. Serial dilutions of hFcRn-His, ranging from 1,000 to 62.5 nM in 1× PBST (pH 6.0), were injected at a rate of 30 µl/min for 120 s, followed by a buffer-only injection for 120 s. The signal was detected at a rate of 10 signals per seconds. Binding constants were determined using the 1:1 binding model in the Biacore T200 Evaluation software (version 3.1). After each binding cycle, the chip was regenerated by injecting 0.5 M arginine (pH 8.0) and 100 mM Tris-HCl (pH 8.0) for 60 s at a rate of 30 µl/min. All experiments were conducted in triplicate.

### Biolayer interferometry (BLI) analysis for rank ordering of on-rate and off-rate

The Octet R8 instrument (Sartorius, Göttingen, Germany) was employed to measure the on-rate at pH 6.0 and the off-rate at pH 7.4 for trastuzumab-Fc variants. Prior to measurement, Octet NTA biosensors were hydrated in distilled water for 10 min using a black 96-well plate. Subsequently, the NTA biosensors were incubated with 40 µg/ml of hFcRn-His for 180 s to facilitate immobilization, followed by baseline measurement in PBS (pH 6.0). Trastuzumab-Fc variants, diluted in PBS (pH 6.0), were bound for 20 s and immediately dissociated by incubation in PBS (pH 7.4) for 5 s. Initial on-rate and off-rate were calculated based on signal measurements during the first 2 s of the association phase and the first 1 s of the dissociation phase from the sensorgrams.

### Pharmacokinetic profile analysis

All animal procedures were conducted following approval from the Institutional Animal Care and Use Committee (IACUC) of the authors’ affiliated institution. To analyze the pharmacokinetic profiles of trastuzumab-Fc variants, including trastuzumab, trastuzumab-PFc29, trastuzumab-DHS, trastuzumab-YML, and trastuzumab-EML, these Fc variants were intravenously injected at a dose of 5 mg/kg into hFcRn transgenic mice (B6.Cg-*Fcgrt*^*tm1Dcr*^ Tg(CAG-FCGRT)276Dcr/DcrJ hemizygous) (*n* = 3). Blood samples were collected from the facial vein at the following time points post-injection: 0.5, 24, 168, 336, 504, 672, 840, 1,008, and 1,176 h. To determine the concentration of trastuzumab-Fc variants in serum samples, 96-well microplates were coated with 4 µg/ml HER2 diluted in 0.05 M Na₂CO₃ (pH 9.6) and blocked with 4% skim milk in 1× PBS. Following four washes of the plates with 180 µl of 0.05% PBST (1× PBS and 0.05% Tween-20), serially diluted mouse serum samples or trastuzumab-Fc variants (used as a standard) were added, and the plates were incubated at room temperature for one hour. The plates were then washed four times with 0.05% PBST, and 50 µl of peroxidase-conjugated AffiniPure F(ab’)₂ Fragment Goat Anti-Human IgG (H + L) was added, followed by incubation for one hour at room temperature. After another four washes, 50 µl of 1-Step Ultra TMB ELISA substrate solution was added, followed by the addition of 50 µl of 2 M H₂SO₄. Absorbance at 450 nm was measured using an Epoch microplate spectrophotometer (BioTek).

### CDC assay

Ramos cells (5 × 10^5^) were incubated in a V-bottom 96 well cell culture plate with or without treatment of rituximab-Fc variants in phenol-free RPMI-1640 medium containing 20% (v/v) human complement sera. The incubation was performed for 3 h at 37 °C in an atmosphere of 5% CO_2_. After incubation, lysed cells were stained using the FITC Annexin V Apoptosis Detection Kit with 7-AAD, followed by washing with Annexin V binding buffer. The cells were then fixed with 6% formaldehyde, incubated at room temperature for 15 min, washed again with Annexin V binding buffer. Fluorescence was analyzed using a FACS Lyric instrument (BD biosciences, Franklin Lakes, NJ, USA), and cytotoxicity was calculated as follows: % Cytotoxicity = (Dead cell population / Total cell population) × 100%] − % Baseline cytotoxicity. In this calculation, the dead cell population represents cells positive for both FITC Annexin V and 7-AAD (FITC⁺/7-AAD⁺), while the total cell population encompasses all cells within the sample. Baseline cytotoxicity accounts for non-specific cell death observed under complement-only conditions (without antibody treatment) and is determined as 100 × (Dead cell population / Total cell population).

### Structural modeling

The structure of YML was predicted using ColabFold v.1.5.5 [35], an open-access protein folding prediction platform based on Alphafold2 [36] with MMseqs2 [37]. To determine the protonation status of Fc and its variants at pH 6.0, the crystal structure of the human wild-type Fc (PDB code: 5JII) [38] and the predicted model of YML were processed by H++ [39] to add or remove hydrogen atoms for relevant amino acid residues. The in silico complex structures of YML or wild-type Fc with hFcRn were constructed using ClusPro 2.0 [40] with default protein-protein docking settings. The hFcRn structure from the crystal complex with Fc-MST-HN (PDB code: 7Q15) [41] was used to generate the in silico complex models. Structural superpositions and molecular interaction illustrations were created using the PyMOL Molecular Graphics System and LigPlot [42], respectively.

## Results

### Designing an Fc engineering strategy with consideration of competitive binding dynamics in hFcRn-mediated recycling

In our prior study, we demonstrated that an engineered Fc variant with two mutations (PFc29: Q311R and M428L), which enhances the pH-selective binding affinity for hFcRn, significantly extended serum half-life in hFcRn transgenic mice and cynomolgus monkeys [17]. However, to identify an Fc variant capable of achieving even greater serum half-life extension than PFc29, we developed a novel engineering strategy focusing on the kinetic aspects of hFcRn–Fc interactions, rather than solely on equilibrium binding affinity. Fc-hFcRn interactions between the hFcRn molecule and the Fc region of IgG antibodies. Recognizing that IgG antibodies are “rescued” by hFcRn during intracellular trafficking and recycling, our approach prioritized optimizing the association and dissociation rates under these conditions to enhance the competitive dynamics of therapeutic antibodies against endogenous IgG.

**Fig. 1:**
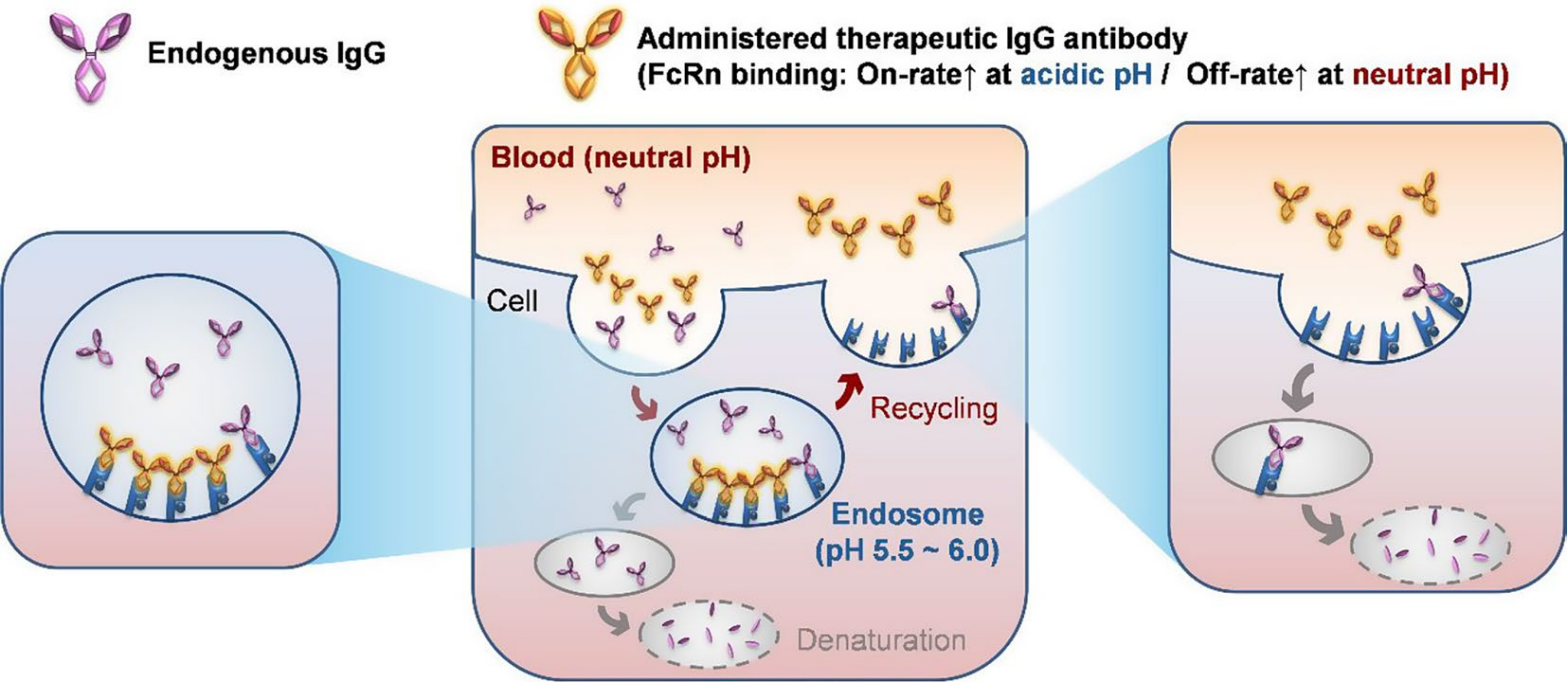
Schematic of the hypothesis for efficient FcRn-mediated recycling. Administered therapeutic IgG antibodies compete with endogenous IgG for limited hFcRn in the endosome. Effective recycling requires therapeutic antibodies to exhibit accelerated hFcRn binding at acidic pH (5.5–6.0) and rapid dissociation at neutral pH, enabling escape from lysosomal degradation, efficient recycling, and prolonged serum persistence

Therapeutically administered monoclonal antibodies circulate at concentrations much lower than the already high levels of endogenous IgG [31, 32, 43], and the spatially restricted availability of hFcRn within endosomes [29, 30] further intensifies this competition. Moreover, the narrow temporal window before lysosomal degradation imposes additional constraints on efficient recycling. To address these spatial and temporal challenges, we hypothesized that Fc variants with faster association rates at acidic endosomal pH, coupled with rapid dissociation at neutral pH, would gain a competitive binding advantage over endogenous IgG, ultimately improving serum half-life (Fig. 1).

### Discovery of Fc variants with accelerated pH-selective association and dissociation kinetics in hFcRn-mediated recycling

**Fig. 2:**
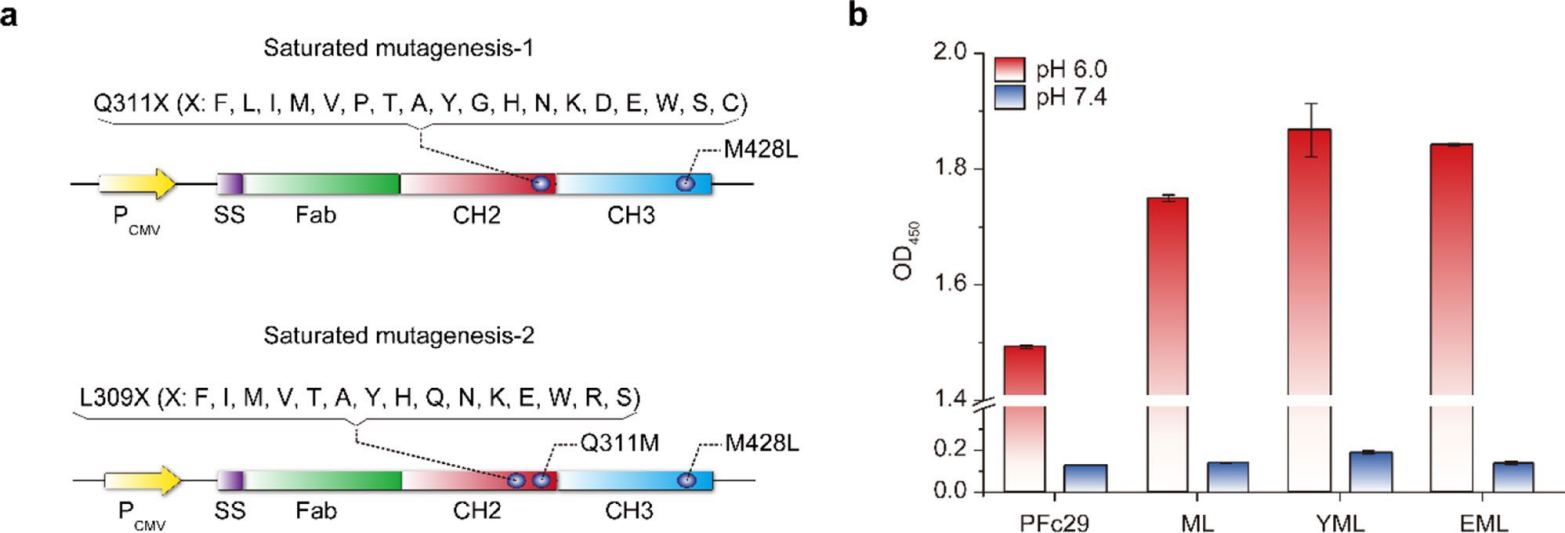
Identification of Fc variants with optimized pH-dependent hFcRn binding profiles. **a** Schematic representation of the focused screening strategy using saturation mutagenesis. In saturation mutagenesis-1, mutations at position Q311 were introduced based on the trastuzumab-M428L scaffold [17] to identify key substitutions enhancing hFcRn binding. Following the identification of the trastuzumab-ML variant, saturation mutagenesis-2 targeted position L309 on the trastuzumab-ML scaffold to further refine binding kinetics, resulting in the discovery of YML and EML variants. **b** Binding profiles of the engineered Fc variants assessed via ELISA. Bar graphs display the binding of Fc variants to hFcRn under mildly acidic (pH 6.0, red) and neutral (pH 7.4, blue) conditions. Trastuzumab-YML and trastuzumab-EML exhibited significantly enhanced hFcRn binding at acidic pH compared to PFc29, while maintaining comparable binding at neutral pH. Error bars represent standard deviations from duplicate experiments

To experimentally validate our hypothesis, we implemented a focused screening strategy aimed at identifying Fc variants with precisely optimized binding kinetics that selectively respond to the pH conditions of endosomal and serum environments, thereby enhancing the efficiency of hFcRn-mediated recycling (Fig. 2a). Our investigation began with residue 311, a pivotal component at the hFcRn-Fc binding interface [10], known to critically influence pH-dependent binding affinity and contribute to the extended serum half-life observed in PFc29 [17]. We systematically introduced 18 single-point mutations into a trastuzumab-Fc variant, intentionally excluding the Gln from wild-type Fc and the Arg mutation from PFc29 to explore alternative substitutions. Among these engineered variants, the ML variant (Q311M/M428L) exhibited markedly enhanced binding to hFcRn at mildly acidic pH, surpassing the binding performance of PFc29. Building on this promising finding, we explored additional substitutions at position 309 in the PFc41 variant, omitting structurally disruptive residues (Pro, Cys), the wild-type Leu, and previously reported mutations (Gly, Asp). This refined approach led to the identification of two standout candidates: YML (L309Y/Q311M/M428L) and EML (L309E/Q311M/M428L): Both demonstrated significantly stronger hFcRn binding in ELISA assays, surpassing PFc29 (Fig. 2b). Further validation using pH-gradient FcRn affinity column analysis revealed that these variants exhibited extended retention times, indicating enhanced hFcRn binding affinity relative to the wild-type Fc (retention times: trastuzumab, 40.64 min (pH 7.65); trastuzumab-EML, 48.00 min (pH 7.97); trastuzumab-PFc29, 50.18 min (pH 8.05); trastuzumab-YML, 51.24 min (pH 8.09)) (Supplementary Fig. 1).

**Fig. 3:**
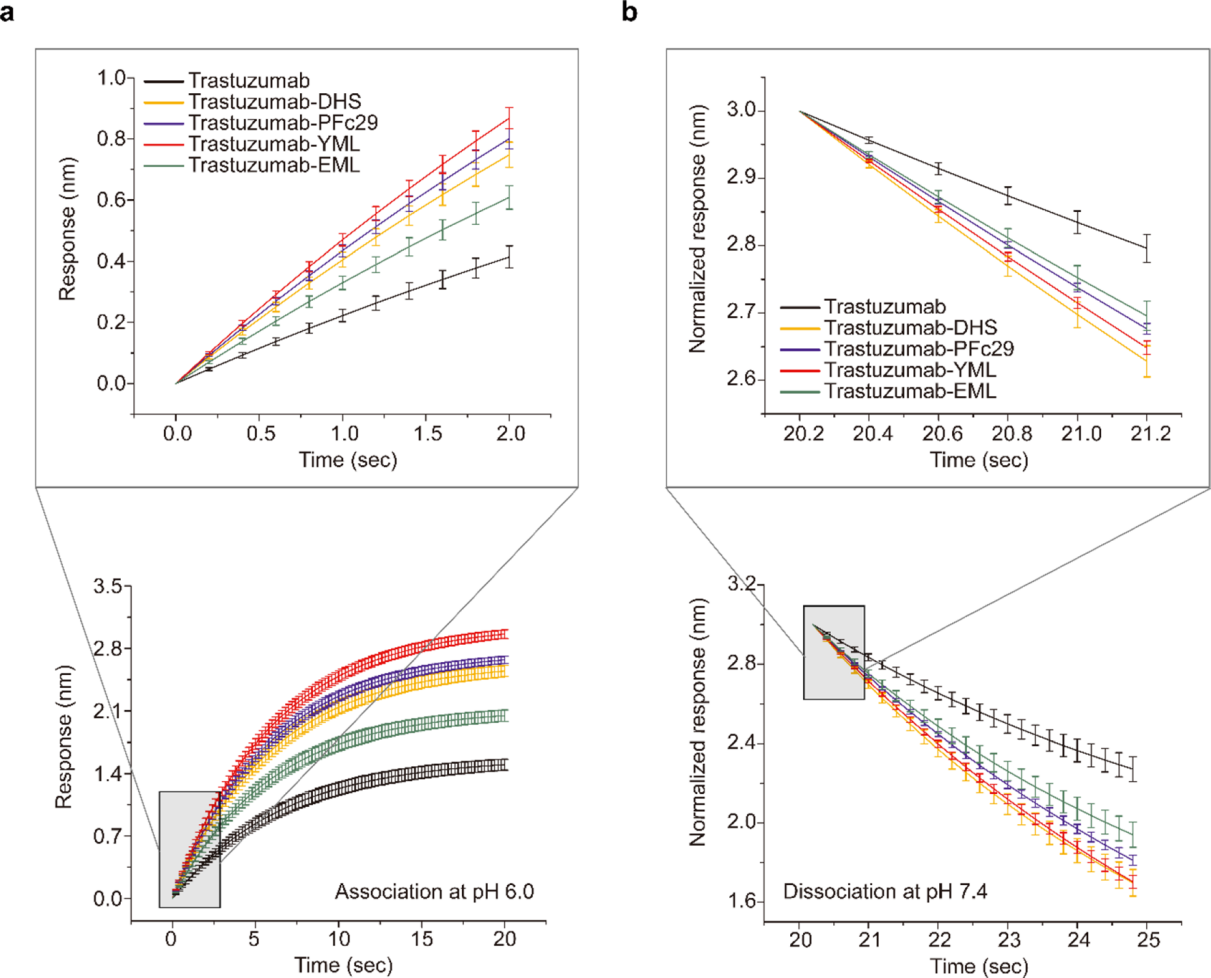
Biolayer interferometry (BLI) analysis of trastuzumab and trastuzumab-Fc variants to evaluate hFcRn binding kinetics. **a** Initial association rates (on-rates) of trastuzumab, trastuzumab-DHS, trastuzumab-PFc29, trastuzumab-YML, and trastuzumab-EML were determined at pH 6.0 using the Octet R8 system. hFcRn-His was immobilized onto Ni-NTA biosensors, and Fc variants diluted in PBS (pH 6.0) were introduced for 20 s. Early association rates were calculated based on the first 2 s of the sensorgram. **b** Initial dissociation rates (off-rates) of trastuzumab and Fc variants were measured at pH 7.4. After the association phase, biosensors were transferred to PBS (pH 7.4) for 5 s, and early dissociation rates were calculated based on the first 1 s of the dissociation phase. Trastuzumab-YML exhibited the fastest on-rate and off-rate among all Fc variants, reflecting its enhanced pH-selective binding kinetics. Error bars represent the standard deviations calculated from quadruplicate experiments

We next investigated the kinetic binding behavior of these Fc variants using BLI on an Octet instrument. We measured initial association rates (ΔR/Δsec) with hFcRn at pH 6.0 and subsequent dissociation rates at neutral pH 7.4 (Fig. 3; Table 1, Supplementary Table 3). This analysis confirmed trastuzumab-YML as the top candidate, showing a 2.1-fold increase in association rate compared to trastuzumab. Regarding dissociation kinetics, trastuzumab-DHS (1.831 ± 0.071-fold) and trastuzumab-YML (1.737 ± 0.125-fold) exhibited the fastest release rates among tested variants, within the experimental error margin. Overall, trastuzumab-YML demonstrated an impressive 1.921-fold increase in both association and dissociation rates relative to trastuzumab, validating its superior pH-selective binding kinetics with hFcRn. Collectively, these findings establish trastuzumab-YML as a candidate capable of rapidly binding to hFcRn in the acidic endosomal environment and quickly disengaging at neutral pH, thereby gaining a competitive advantage over serum IgG and potentially enhancing serum half-life.

**Table 1:** Relative initial on- and off-rates for hFcRn binding of trastuzumab and trastuzumab Fc variants at the initial phases of association (pH 6.0) and dissociation (pH 7.4)

	Association at pH 6.0		Dissociation at pH 7.4		Average of relative on- and off-rate	Rank
	Relative on-rate	R^2^	Relative off-rate	R^2^		
Trastuzumab	1.000±0.000	0.9982	1.000±0.000	0.9995	1.000±0.000	5
Trastuzumab-DHS	1.809±0.054	0.9978	1.831±0.071	0.9994	1.820±0.011	2
Trastuzumab-PFc29	1.940±0.094	0.9977	1.598±0.123	0.9996	1.769±0.171	3
Trastuzumab-YML	2.105±0.119	0.9979	1.737±0.125	0.9996	1.921±0.184	1
Trastuzumab-EML	1.472±0.067	0.9978	1.497±0.084	0.9994	1.484±0.012	4

### Trastuzumab-Fc variant has increased binding affinity to hFcRn and comparable druggability

The binding affinities of trastuzumab-Fc variants to hFcRn were quantitatively assessed under mildly acidic conditions (Table 2). Among the engineered variants, trastuzumab-YML demonstrated a remarkable five-fold increase in binding affinity compared to wild-type trastuzumab. Notably, trastuzumab-YML exhibited approximately a two-fold higher binding affinity to hFcRn than trastuzumab-PFc29, an Fc variant identified in our prior studies to provide superior circulating half-life over trastuzumab variants incorporating YTE and LS mutations in cynomolgus monkeys. Furthermore, trastuzumab-YML also surpassed trastuzumab-DHS, developed by Georgiou’s group, which previously exhibited an extended circulating half-life relative to trastuzumab-Fc variants containing YTE and LS in hFcRn transgenic mice [16]. Trastuzumab-EML, another promising variant, displayed a 2.6-fold enhancement in hFcRn binding affinity compared to wild-type trastuzumab (Table 2, Supplementary Fig. 2).

**Table 2:** Association and dissociation rate constants (k_a_ and k_d_), and equilibrium dissociation constants (K_D_) in the interaction of trastuzumab and trastuzumab-Fc variants with hFcRn at pH 6.0

	k_a_ (M^− 1^s^− 1^)	k_d_ (s^− 1^)	K_D_ (nM)
Trastuzumab	Steady state		4360
Trastuzumab-DHS	3.57 × 10^4^	0.0645	1800
Trastuzumab-PFc29	3.98 × 10^4^	0.0721	1810
Trastuzumab-YML	6.32 × 10^4^	0.0555	878
Trastuzumab-EML	2.85 × 10^4^	0.0475	1670

In addition to their enhanced binding characteristics, trastuzumab-Fc variants incorporating engineered mutations retained physicochemical properties comparable to those of wild-type trastuzumab, including thermostability, aggregation propensity, and glycan profiles (Table 3, Supplementary Fig. 3–5). In the thermofluor assay, the melting temperatures (T_m_) of trastuzumab and the trastuzumab Fc variants were determined by monitoring the increase in fluorescence associated with protein unfolding as the temperature increased [44–46]. Both identified Fc variants, trastuzumab-YML and trastuzumab-EML, demonstrated comparable thermostabilities to trastuzumab, trastuzumab-PFc29, and trastuzumab-DHS (69.03 ± 0.14 °C for trastuzumab, 68.74 ± 0.49 °C for trastuzumab-DHS, 69.00 ± 0.10 °C for trastuzumab-PFc29, 67.57 ± 0.05 °C for trastuzumab-YML, and 67.30 ± 0.22 °C for trastuzumab-EML) (Table 3 and Supplementary Fig. 5). In addition, immunogenicity prediction analysis using The Immune Epitope Database (IEDB) for the 27 most prevalent HLA types indicated that peptides containing L309Y/Q311M in YML and L309E/Q311M in EML had lower percentile ranking scores for predicted binding affinity to MHC class II than those derived from the wild-type Fc or PFc29. Thus, these Fc mutations did not significantly increase the predicted risk of immunogenicity compared to the wild-type Fc (Supplementary Fig. 6) [17]. Together, these findings underscore the potential of trastuzumab-YML and trastuzumab-EML as robust candidates for therapeutic development, offering enhanced hFcRn binding characteristics while maintaining favorable druggability profiles.

**Table 3:** Thermostabilities (T_m_) of trastuzumab and trastuzumab-Fc variants

Name of Fc variants	Apparent T_m_ (°C)	pI	Mw (kDa)
Trastuzumab	69.03±0.14	8.76	148.84
Trastuzumab-DHS	68.74±0.49	8.55	148.82
Trastuzumab-PFc29	69.00±0.10	8.67*	148.87
Trastuzumab-YML	67.57±0.05	8.75	148.92
Trastuzumab-EML	67.30±0.22	8.58	148.85

### YML Fc variant dramatically extends IgG serum half-life over wild-type and pre-existing Fc variants

**Fig. 4:**
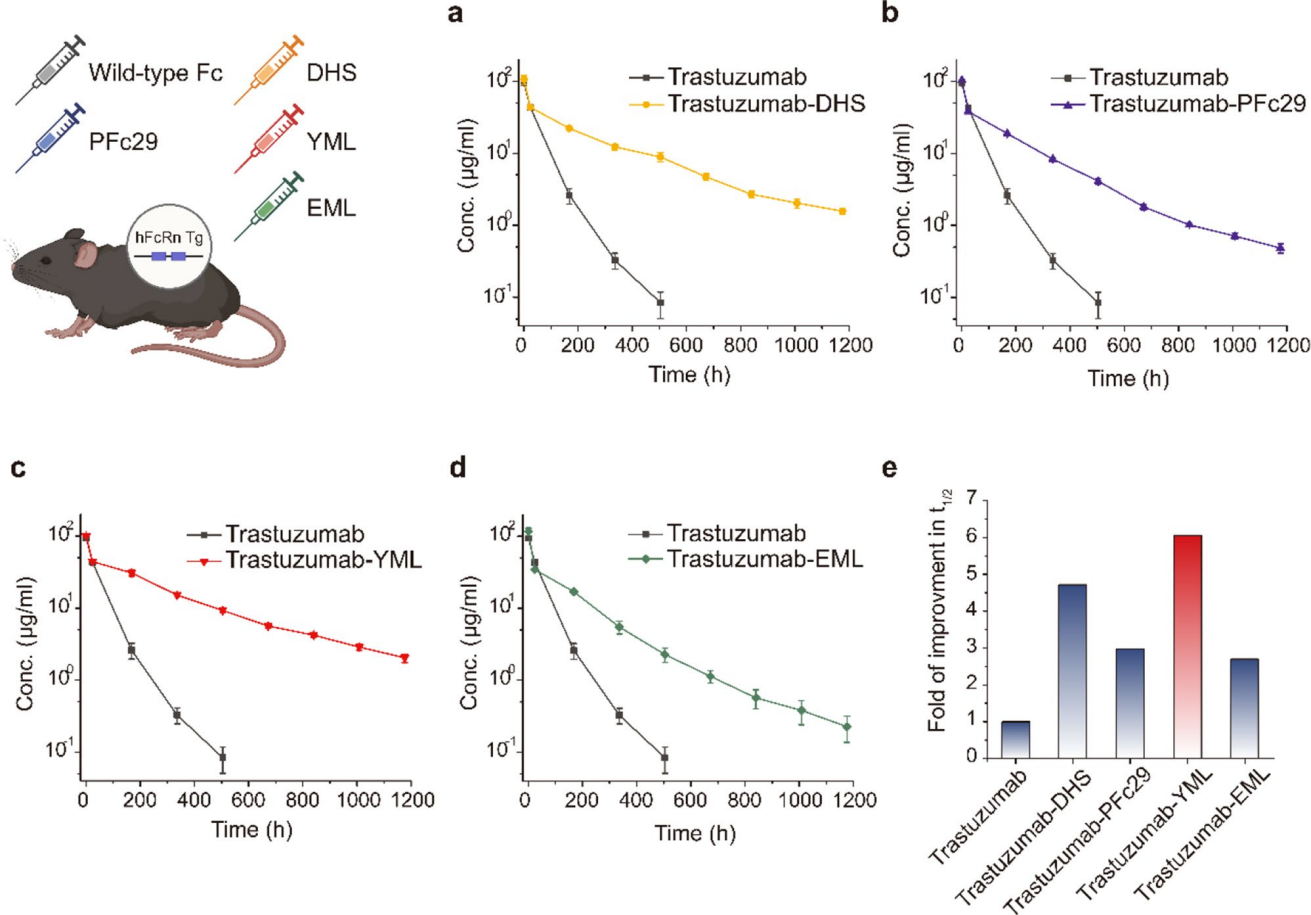
Pharmacokinetic analysis of trastuzumab and trastuzumab-Fc variants in hFcRn transgenic mice. **a– d** Serum concentrations of trastuzumab (black line) and trastuzumab-Fc variants, including trastuzumab-DHS **a** (yellow), trastuzumab-PFc29 **b** (purple), trastuzumab-YML **c** (red), and trastuzumab-EML **d** (green), were measured over a 7-week period following intravenous administration (5 mg/kg, *n* = 3). Error bars represent standard deviations. Trastuzumab-YML demonstrated the most extended serum persistence among the Fc variants, highlighting its superior pharmacokinetic properties. **e** Fold improvement in serum half-life (t_₁/₂_) of trastuzumab-Fc variants relative to wild-type trastuzumab. Trastuzumab-YML achieved the most significant extension in t_₁/₂_, surpassing other variants, including trastuzumab-DHS, trastuzumab-PFc29, and trastuzumab-EML, further emphasizing its potential as a next-generation Fc-engineered therapeutic

To evaluate the pharmacokinetic profiles of YML and EML Fc variants, we conducted in vivo studies using hFcRn transgenic mice (B6.Cg-*Fcgrt*^*tm1Dcr*^ Tg(CAG-FCGRT)276Dcr/DcrJ hemizygous). Mice were intravenously administered trastuzumab-Fc variants at 5 mg/kg via the tail vein (*n* = 3), and serum samples were collected over a 7-week period to determine circulating levels of each variant. Pharmacokinetic analysis revealed that while trastuzumab-EML exhibited reduced pharmacokinetic parameters compared to trastuzumab-PFc29, trastuzumab-YML demonstrated an exceptional serum half-life of approximately 324.45 h. This represents a 6.1-fold increase compared to wild-type trastuzumab (undetectable after 3 weeks) and a two-fold improvement over trastuzumab-PFc29, the parent Fc variant (Fig. 4; Table 4). Notably, trastuzumab-YML extended the serum half-life by approximately 71 h compared to trastuzumab-DHS, a variant that had previously demonstrated superior pharmacokinetics compared to widely studied Fc-engineered variants such as Xencor’s LS variant [14] and AstraZeneca’s YTE variant [47]. Specifically, the serum half-life values were as follows: trastuzumab-PFc29, 159.75 h; trastuzumab-DHS, 252.69 h; and trastuzumab-YML, 324.45 h. Furthermore, the total drug exposure over time (AUC_inf_) for trastuzumab-YML was calculated as 16,684.21 µg/ml×h, reflecting a marked improvement over trastuzumab-DHS, which achieved an AUC_inf_ of 13,739.94 µg/ml×h. These enhanced pharmacokinetic characteristics of trastuzumab-YML strongly correlate with the initial kinetic analysis of association and dissociation rates between the trastuzumab-Fc variants and hFcRn. The rank order of average relative on- and off-rates across two distinct pH conditions (pH 6.0 and pH 7.4) aligns precisely with the observed pharmacokinetic profiles (YML > DHS > PFc29 > EML > wild-type Fc) (Table 1). This clear concordance highlights the critical role of pH-selective binding kinetics in dictating serum persistence, underscoring trastuzumab-YML as a transformative advancement in Fc engineering for therapeutic antibody optimization.

**Table 4:** Noncompartmental pharmacokinetic parameters for trastuzumab and trastuzumab-Fc variants in hFcRn mouse (Tg276 hemizygous)

	Trastuzumab	Trastuzumab -DHS	Trastuzumab -PFc29	Trastuzumab -YML	Trastuzumab -EML
t_1/2_(h)	53.55 ± 3.07	252.69 ± 16.34	159.75 ± 6.26	324.45 ± 19.18	144.53 ± 0.69
C_max_(µg/ml)	94.35 ± 4.82	105.31 ± 11.90	103.24 ± 2.91	99.90 ± 1.26	116.48 ± 13.94
AUC_last_(µg/ml×h)	3843.92 ± 204.80	13164.82 ± 774.01	9617.51 ± 67.49	15717.56 ± 632.22	8019.11 ± 506.04
AUC_inf_(µg/ml×h)	3850.57 ± 207.35	13739.94 ± 828.64	9729.08 ± 86.83	16684.21 ± 800.83	8066.59 ± 524.99
AUC_%_	0.17 ± 0.068	4.18 ± 0.50	1.15 ± 0.19	5.76 ± 0.96	0.58 ± 0.21
CL_inf_(ml/h)	0.0013 ± 6.8 × 10^− 5^	0.00037 ± 2.2 × 10^− 5^	0.00051 ± 4.2 × 10^− 6^	0.0003 ± 1.43 × 10^− 5^	0.00062 ± 4.21 × 10^− 5^

t_1/2_: terminal half-life (β-phase); C_max_: maximum concentration; AUC_last_: area under the curve from dosing to the last measurable concentration; AUC_inf_: area under the curve from administration to infinity; AUC_%_: percentage of the extrapolated AUC relative to the total AUC; CL_inf_: total body clearance. Dose and route: single i.v. bolus at 5 mg/kg (*n* = 3) for mouse

### YML Fc variant retains hFcγR binding while significantly enhancing human C1q binding and target cell clearance

**Fig. 5:**
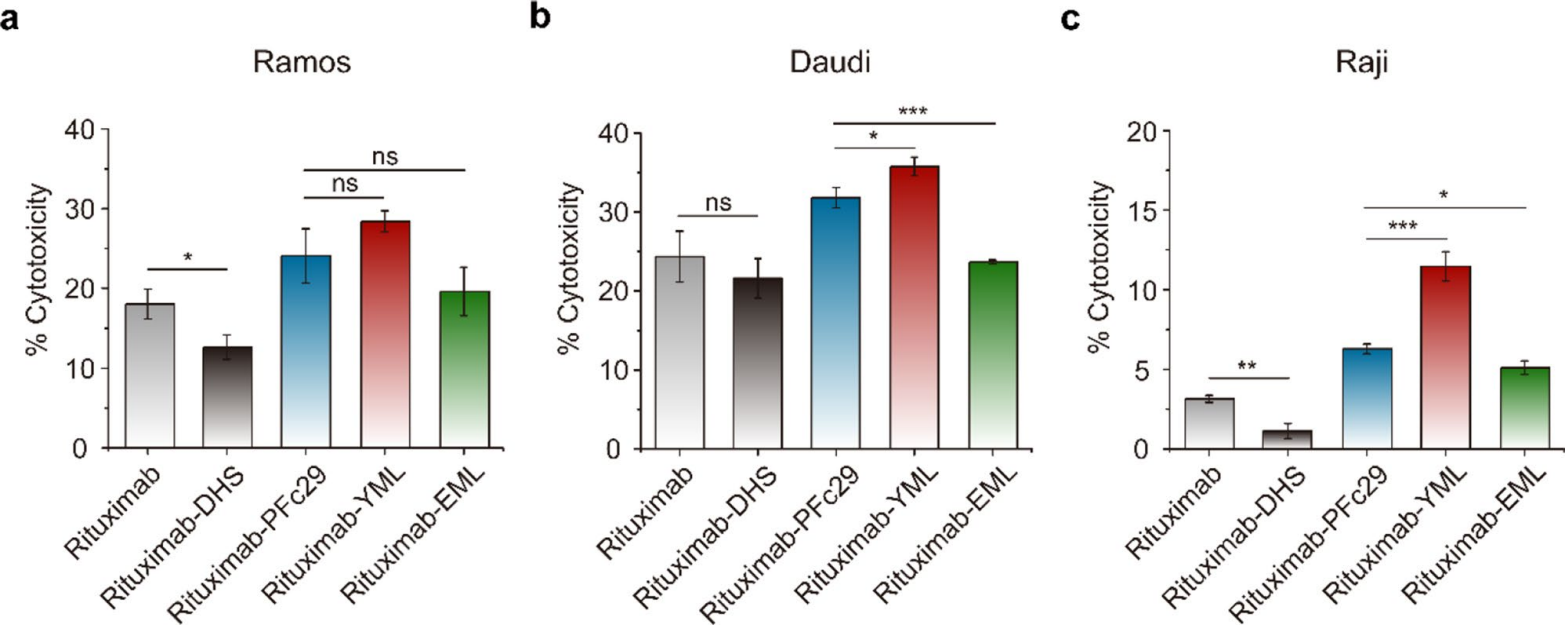
Complement-dependent cytotoxicity (CDC) activities of rituximab and rituximab-Fc variants. **a– c** CDC activity was measured for rituximab and its Fc-engineered variants (rituximab-DHS, rituximab-PFc29, rituximab-YML, and rituximab-EML) using three CD20-overexpressing lymphoma B cell lines: Ramos **a**, Daudi **b**, and Raji **c**. Cytotoxicity was assessed based on the activation of the complement cascade in the presence of human serum. Rituximab-YML consistently exhibited the highest CDC activity across all cell lines, outperforming other Fc variants, including rituximab-PFc29 and rituximab-DHS. Error bars represent standard deviations derived from triplicate experiments. Statistical significance was evaluated as follows: *p* > 0.05 (ns, not significant), *p* ≤ 0.05 (*), *p* ≤ 0.01 (**), *p* ≤ 0.001 (***)

Mutations introduced into the Fc region to enhance hFcRn binding and improve pharmacokinetic profiles require careful validation, as they may also alter Fc-mediated effector functions, both positively and negatively [48–50]. We evaluated the binding affinities of trastuzumab-Fc variants (-YML and -EML) to hFcγRs expressed on leukocytes, including hFcγRI, hFcγRIIa-131H, hFcγRIIa-131R, hFcγRIIb, hFcγRIIIa-158V, and hFcγRIIIa-158F. These receptors mediate antibody effector functions such as antibody-dependent cellular cytotoxicity (ADCC) and antibody-dependent cellular phagocytosis (ADCP). Additionally, we assessed hC1q binding, which initiates CDC. The results demonstrated that trastuzumab-Fc variants retained binding affinities to all hFcγRs comparable to wild-type trastuzumab (Supplementary Fig. 7a–f). Notably, trastuzumab-YML exhibited significantly enhanced hC1q binding compared to trastuzumab and other Fc-engineered variants (Supplementary Fig. 7g). To further investigate the functional implications of enhanced hC1q binding, we prepared rituximab (anti-CD20) harboring the identified Fc variants (Supplementary Fig. 8–9) and analyzed CDC activity using CD20-overexpressing lymphoma B cell lines (Ramos, Daudi, Raji) as target cells. This approach was employed instead of SK-BR-3 cells, which are known to suppress CDC activity through overexpression of complement regulatory proteins such as CD55 and CD59 [51, 52]. CDC assays revealed that rituximab-YML demonstrated significantly improved cytotoxicity compared to wild-type rituximab, showing 28.4 ± 1.3% for Ramos, 35.8 ± 1.2% for Daudi, and 11.5 ± 0.9% for Raji (Fig. 5). Importantly, rituximab-YML surpassed rituximab-PFc29, a previously identified variant with enhanced CDC activity [17], which demonstrated 24.1 ± 3.4% for Ramos, 31.8 ± 1.3% for Daudi, and 6.3 ± 0.3% for Raji. Rituximab-EML showed comparable or slightly enhanced CDC activity relative to rituximab, whereas rituximab-DHS exhibited reduced CDC activity (12.7 ± 1.5% for Ramos, 21.6 ± 2.5% for Daudi, and 1.1 ± 0.5% for Raji). These findings highlight the dual benefits of the YML Fc variant, which not only extends serum half-life but also significantly enhances CDC activity, suggesting its potential utility in therapeutic antibodies requiring robust target cell clearance, such as those for cancer immunotherapy.

### Structure-based molecular insights into the enhanced pH-dependent interaction of YML

**Fig. 6:**
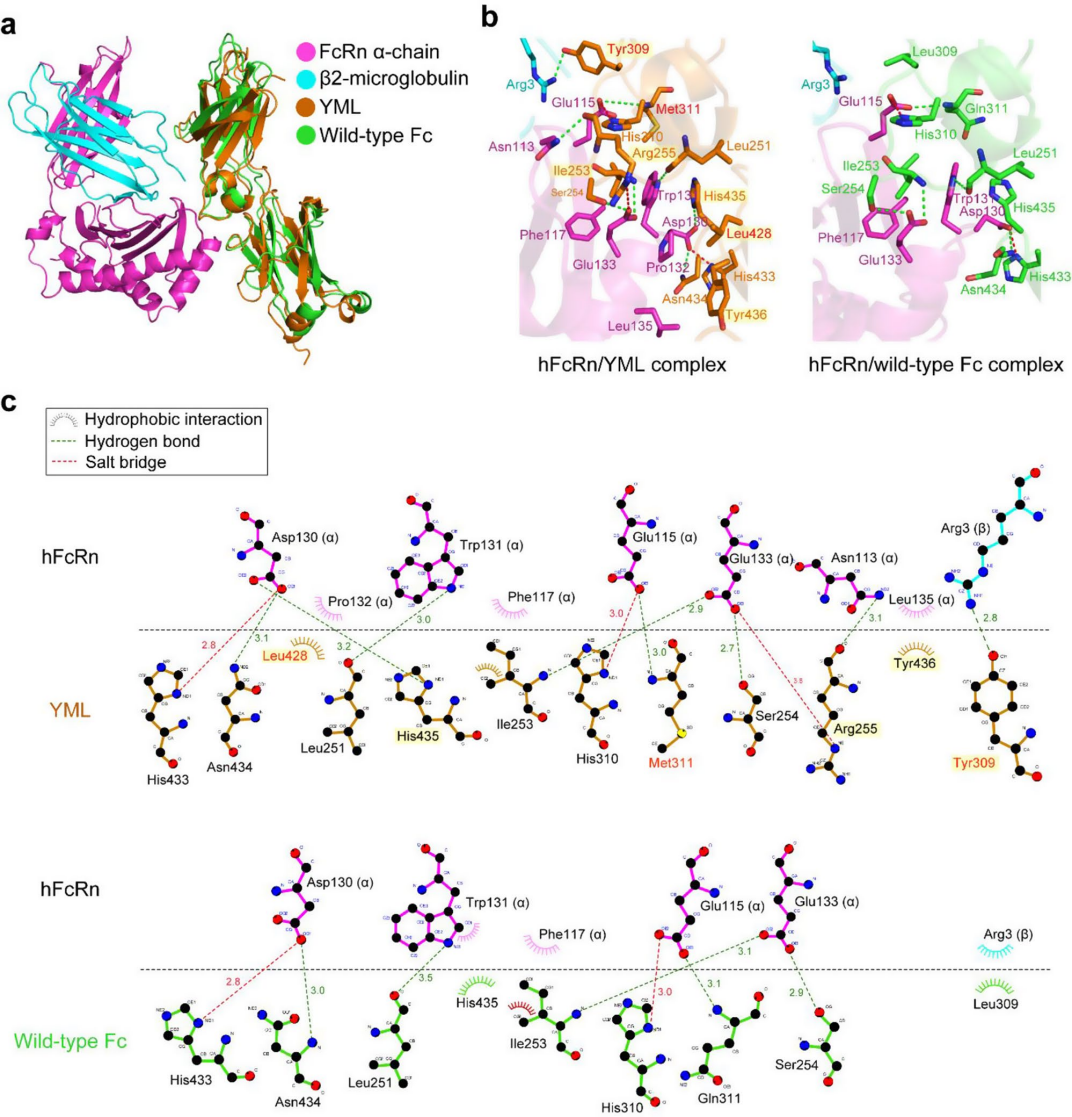
Structural insights into the enhanced pH-dependent interaction of the YML Fc variant. **a** Superimposed complex model structures of hFcRn/YML and hFcRn/wild-type Fc, illustrating the conformational differences. The hFcRn α-chain, β2-microglobulin, YML, and wild-type Fc are shown in magenta, cyan, brown, and green, respectively. The structures were superimposed based on the hFcRn α-chain and β2-microglobulin to highlight conformational shifts induced by YML mutations. **b** Molecular interactions within the hFcRn/YML and hFcRn/wild-type Fc complex models visualized using PyMOL. Notable interactions unique to YML include hydrophobic and electrostatic contacts between L428, R255, and Y436 of YML and residues of the hFcRn α-chain (P132, L135, N113, and E133). **c** Detailed molecular interactions depicted with LigPlot. Salt bridges and hydrogen bonds are indicated by red and green dotted lines, respectively. Additional interactions unique to YML, such as those involving Y309, M311, and R255, contribute to its enhanced binding kinetics under acidic conditions, compared to wild-type Fc

To elucidate the molecular basis of YML’s enhanced pH-dependent interaction with hFcRn, we constructed in silico complex models of hFcRn/YML and hFcRn/wild-type Fc using ClusPro 2.0. The interaction residues identified in the hFcRn/wild-type Fc complex (Fig. 6a–c) closely correlate with those determined in full-length hIgG1 and hFcRn using HDX-MS spectrometry [53], underscoring the reliability of the in silico models. Analysis of the hFcRn/YML complex revealed that the three mutations in YML (L309Y/Q311M/M428L) induced a notable conformational shift in the Fc region, bringing the Fc closer to hFcRn (Fig. 6a) and creating distinct molecular interactions absent in the wild-type Fc (Fig. 6b and c). Specifically, three additional residues in YML—L428, R255, and Y436—formed novel interactions with hFcRn. L428 and Y436 engaged in hydrophobic interactions with P132 and L135 of the hFcRn α-chain, respectively, while R255 established electrostatic interactions with N113 and E133 of the α-chain (Fig. 6b and c). These interactions likely enhance YML’s binding affinity for hFcRn under mildly acidic conditions (pH 6.0). Notably, the hydrophobic interactions previously observed between hFcRn and the wild-type Fc (L309 of wild-type Fc–R3 of hFcRn β2-microglobulin and H435 of wild-type Fc–W131 of hFcRn α-chain) were replaced in YML by electrostatic interactions (Y309 of YML–R3 of hFcRn β2-microglobulin and H435 of YML–D130 of hFcRn α-chain) (Fig. 6b and c). Given the central role of histidine residues in pH-dependent Fc-hFcRn interactions, the electrostatic interaction involving H435 likely enhances YML’s binding affinity under acidic conditions. Furthermore, the pH effect is modulated by shifts in electrostatic interactions [54], which influence Fc’s conformational dynamics, particularly within residues 250–255 [20]. These shifts, induced by R255 of YML, which interacts with N113 and E133 of the hFcRn α-chain, appear to stabilize the Fc–hFcRn complex and contribute significantly to YML’s superior pH-selective binding affinity.

## Discussion

Extending the serum half-life of therapeutic antibodies has long been rooted in the mechanistic understanding of pH-dependent interactions between the IgG Fc region and the FcRn. This interaction allows IgG antibodies internalized via pinocytosis to bind FcRn under acidic endosomal conditions and dissociate at neutral pH in the bloodstream, facilitating efficient recycling and protection from lysosomal degradation. Leveraging this mechanism, Fc variants such as LS and YTE have been widely adopted, with applications in antibody and Fc-fusion protein design. These variants are integral components of several FDA-approved therapeutics, including Beyfortus^®^, approved in 2023, underscoring their clinical relevance [22].

Recently, the DHS variant has demonstrated a refined approach to half-life extension. Although DHS binds less strongly to hFcRn under endosomal pH conditions compared to YTE and LS, its minimal binding at neutral pH enables efficient antibody release into circulation. This characteristic results in significantly enhanced serum persistence in hFcRn transgenic mice compared to YTE and LS [16]. These findings highlight the importance of achieving strong hFcRn binding under acidic conditions while ensuring rapid dissociation at physiological pH to optimize pharmacokinetics. Furthermore, studies have shown that Fc variants with consistently strong hFcRn binding under both acidic and neutral pH conditions, such as N434W, fail to achieve meaningful improvements in serum half-life, underscoring the critical necessity for exceptionally fine-tuned pH selectivity [18].

Building upon these insights, our study moves beyond equilibrium binding affinity to address the spatial and temporal constraints inherent in the endosomal environment. Within endosomes, hFcRn availability is limited to approximately 50 µM, while serum IgG levels exceed 100 µM [29–32], creating intense competition for binding. Therapeutic antibodies that fail to bind hFcRn within this narrow timeframe are rapidly degraded in lysosomes. To address this challenge, we hypothesized that an Fc variant optimized for rapid hFcRn association under acidic pH and swift dissociation at neutral pH could outcompete endogenous IgG, thereby enhancing recycling efficiency and extending serum persistence. To explore the validity of this hypothesis, we performed comprehensive site-directed saturation mutagenesis based on the previously developed PFc29 variant, focusing on optimizing binding kinetics. This approach led to the identification of YML, an engineered Fc variant that demonstrated superior hFcRn engagement kinetics under acidic conditions and efficient release at neutral pH. Antibodies incorporating YML exhibited extended serum half-life compared to DHS variants, driven by rapid binding to the limited number of hFcRn molecules within endosomes and effective release back into circulation. These findings highlight the critical role of binding kinetics, alongside equilibrium constants, in optimizing hFcRn-mediated recycling. Through site-directed saturation mutagenesis of the PFc29 variant, we identified YML as a novel Fc variant with optimized binding kinetics. YML exhibited rapid association with hFcRn under acidic conditions and efficient release at neutral pH, leading to significantly prolonged serum half-life compared to DHS and other Fc variants. In contrast, EML, despite improved binding affinity (K_D_) at acidic pH relative to PFc29, exhibited slower initial association rates and inferior pharmacokinetics in vivo. These findings reaffirm the importance of not only equilibrium constants but also binding kinetics in hFcRn-mediated recycling. The rank order of pharmacokinetics among tested variants—YML > DHS > PFc29 > EML > wild-type Fc—directly correlated with their kinetic profiles, emphasizing the critical role of rapid on- and off-rates (Figs. 3 and 4e; Tables 1 and 2, and 4). While consistent with Borrok et al.‘s hypothesis that exceeding a threshold affinity at neutral pH impairs antibody dissociation [55], these findings advance this understanding by highlighting the critical role of rapid on- and off-rates. These findings advance this understanding by highlighting the critical role of rapid on- and off-rates. YML’s success further demonstrates the necessity of maintaining affinity below this threshold while optimizing kinetic parameters to maximize serum persistence.

Structural modeling revealed molecular insights into YML’s enhanced pH-dependent binding kinetics. Mutations in YML (L309Y/Q311M/M428L) induced a conformational shift in the Fc region, facilitating unique hydrophobic and electrostatic interactions with hFcRn residues, including P132, L135, N113, and E133, which were absent in the wild-type Fc. These interactions likely stabilize the Fc–hFcRn complex under acidic conditions, enhancing YML’s association kinetics. Histidine residues, pivotal in pH-dependent hFcRn binding, contribute significantly to these interactions, particularly through H435 of YML. Additionally, R255 introduced in YML creates new electrostatic interactions with N113 and E133 of hFcRn, further stabilizing the complex and enhancing pH-specific binding affinity. While these structural insights provide compelling mechanistic explanations, complementary studies, including Cryo-EM or X-ray crystallography, combined with real-time binding assays such as FRET, could offer deeper insights into YML’s dynamic molecular interactions.

Beyond extending serum half-life, YML demonstrated superior Fc-mediated effector functions. Fc mutations can inadvertently alter effector activities, as exemplified by YTE mutations, which reduce hC1q binding and compromise CDC [56]. In contrast, YML not only retained hFcγRs binding comparable to wild-type Fc but also exhibited significantly enhanced hC1q binding, leading to superior CDC activity. Despite the absence of mutations within known hC1q-binding epitopes (D270, K322, P329, and P331) [57, 58], structural changes in YML’s CH2 domain likely improve interactions with hC1q’s hexameric globular heads. These dual benefits of enhanced serum persistence and target cell clearance establish YML as a versatile Fc variant for therapeutic applications requiring sustained efficacy and improved effector function.

## Conclusions

In conclusion, YML represents a transformative advancement in Fc engineering, combining extended serum half-life with enhanced CDC-mediated clearance. These attributes position YML as an ideal candidate for antibodies targeting indications that require prolonged drug presence, such as cancer and autoimmune diseases, as well as for prophylactic antibody development. The YML variant can also be adapted to other IgG subclasses (IgG2, IgG3, and IgG4), each of which possesses unique effector functions and pharmacokinetic profiles. Furthermore, the design principles applied in the development of YML, namely the fine-tuning of pH-dependent FcRn binding kinetics, are broadly applicable to other FcRn-interacting biologics. These include Fc-fusion proteins [59] such as VEGFR-Fc [60], IL-1R-Fc [61], EPO-Fc [62], and CTLA4-Fc [63], as well as albumin-fusion proteins [64] and sweeping antibodies [65, 66] developed for efficient antigen clearance. Collectively, these findings deepen our understanding of FcRn-mediated recycling and establish a robust framework for the rational design of next-generation antibody therapeutics with optimized pharmacokinetics and therapeutic efficacy.

## Electronic Supplementary Material

Below is the link to the electronic supplementary material.

Supplementary Material 1

## Data Availability

The datasets generated during and/or analyzed during the current study are available from the corresponding author on reasonable request.
