# Supplementary material for: Engineering FcRn binding kinetics dramatically extends antibody serum half-life and enhances therapeutic potential
Source: J Biol Eng. 2025 Apr 18;19:35. doi: 10.1186/s13036-025-00506-y (PMC12007268; doi:10.1186/s13036-025-00506-y)
Supplement: Supplementary file 1 — Supplementary Material 1 [file 13036_2025_506_MOESM1_ESM.pdf]

SUPPLEMENTARY INFORMATION

Engineering FcRn Binding Kinetics Dramatically Extends Antibody Serum Half-Life and Enhances Therapeutic Potential

Sanghwan Ko[1,†], Migyeong Jo[2,3,†], Munsu Kyung[1,4], Wonju Lee[1,4,††], Woo Hyung Ko[3], Jung-Hyun Na[5], Youn Seo Chun[5], Byoung Joon Ko[5], and Sang Taek Jung[2,3,6,*]

[1]*Department of Biomedical Sciences, Graduate School, Korea University, Seongbuk-gu, Seoul, 02841, Republic of Korea*

[2]*Institute of Chemical Processes, Seoul National University, Gwanak-gu, Seoul, 08826, Republic of Korea*

[3]*Department of Chemical and Biological Engineering, College of Engineering, Seoul National University, Gwanak-gu, Seoul, 08826, Republic of Korea*

[4]*BK21 Graduate Program, Department of Biomedical Sciences, Korea University College of Medicine, Seoul, 02841, Republic of Korea*

[5]*School of Biopharmaceutical and Medical Science, Sungshin Women's University, Gangbuk-gu, Seoul, 01133, Republic of Korea*

[6]*Interdisciplinary Program for Bioengineering, Seoul National University, Seoul, 08826, Republic of Korea*

[†]These authors contributed equally: Sanghwan Ko and Migyeong Jo

††Current affiliation: Manufacturing Science & Technology Team, Manufacturing Science Group2, Samsung Bioepis, Incheon, Republic of Korea

***Correspondence**: *Sang Taek Jung (stjung@snu.ac.kr)

Supplementary Materials and Methods

Construction of plasmids

All plasmids and primers used in this study are summarized in Supplementary Table 1 and Supplementary Table 2. The heavy chains of trastuzumab-ML, -YML, -EML genes were generated by assembly PCR using two fragments (fragment #1 and fragment #2). Fragment #1, encoding the VH-CH1 region of trastuzumab, was amplified using the proper primer set SHK#51/SHK#52 and a template plasmid (pMAZ-IgH-GlycoT) [1]. For fragment #2, each Fc variant was amplified using the following primer pairs: SHK#53/SHK#54 for ML, SHK#61/SHK#56 for YML, and SHK#62/SHK#56 for EML. The fragments were assembled using primers SHK#51/SHK#56. The trastuzumab-DHS variant gene was synthesized by Genscript (Scotch Plains, NJ, USA) and amplified using the primer pair SHK#57/SHK#58. The assembled PCR products and amplified DNA fragments were then ligated into the pMAZ-IgL vector [1] digested with *Bss*HII and *Xba*I restriction endonuclease sites, to generate pMAZ-IgH-trastuzumab-ML, pMAZ-IgH-trastuzumab-YML, pMAZ-IgH-trastuzumab-EML, and pMAZ-IgH-trastuzumab-DHS plasmids. The heavy chains of rituximab-DHS, -YML and -EML genes were similarly constructed by assembly PCR using two fragments (fragment #1 and fragment #2). Fragment #1, encoding the VH-CH1 region of rituximab, was amplified using the primer set SHK#59/SHK#60 and a template plasmid (pMAZ-IgH-rituximab [2]). For fragment #2, each Fc variant gene (DHS, YML and EML) was amplified using the primer pair SHK#55/SHK#56 and assembled with

primers SHK#59/SHK#56. Assembled PCR products were ligated into pMAZ-IgL [1] vector digested with *Bss*HII and *Xba*I restriction endonucleases, resulting in pMAZ-IgH-rituximab-DHS, pMAZ-IgH-rituximab-YML and pMAZ-IgH-rituximab-EML plasmids. To construct pMAZ-HER2-His, the HER2-His encoded gene (uniprot code: P04626) was synthesized by Genscript (Scotch Plains, NJ, USA) and ligated into the pMAZ-IgL vector using *Bss*HII and *Xba*I restriction endonucleases. All ligation products were transformed into *Escherichia coli* Jude1 (F′ [Tn*10(*Tet^{r}*)* proAB^{+} *lacI^{q}* *Δ(lacZ)*M15*]* *mcrA* *Δ(mrr-hsdRMS-mcrBC)* 80d*lac*ZΔM15 Δ*lacX74* *deoR* *recA1* *araD139* Δ *(ara leu)7697* *galU* *galK* *rpsL* *endA1* *nupG*) [3].

Expression and purification of IgG antibody-Fc variants

An IgG heavy chain plasmid (pMAZ-IgH-GlycoT, pMAZ-IgH-trastuzumab-PFc29, pMAZ-IgH-trastuzumab-DHS, pMAZ-IgH-trastuzumab-YML, pMAZ-IgH-trastuzumab-EML, pMAZ-IgH-rituximab, pMAZ-IgH-rituximab-PFc29, pMAZ-IgH-rituximab-DHS, pMAZ-IgH-rituximab-YML, or pMAZ-IgH-rituximab-EML) was co-transfected with an IgG light chain plasmid (pMAZ-IgL-GlycoT or pMAZ-IgL-rituximab) into Expi293F cells using PEI-Max. Following incubation in GIBCO FreeStyle™ 293 expression medium at 37°C with 8% CO_2 for 7 days, the culture was centrifuged at 2,000 × g for 10 min. The supernatants were mixed with 40 ml of 25× PBS per liter of culture, filtered through a 0.2-µm bottle-top filter, and combined with 1 ml of Protein A agarose. After overnight incubation at 4°C, the resin was transferred to a polypropylene column and rinsed twice with 10 mL of 1× PBS. The bound

trastuzumab- and rituximab-Fc variants were eluted using 3 mL of 100 mM glycine-HCl (pH 2.7). The eluate was immediately neutralized by mixing with 1 mL of 1 M Tris (pH 8.0) in a collection tube. Subsequently, the buffer was exchanged with 1× PBS, and the protein samples were concentrated using Amicon Ultra-4 spin columns with a 3-kDa cutoff filter. The purity of the proteins was analyzed on 4–15% resolving SDS-PAGE gels. For animal model injection, the buffer was exchanged with endotoxin-free DPBS using the NGC chromatography system (Bio-Rad, Hercules, CA, USA).

FcRn affinity column chromatography analysis

Antibody samples containing 150 µg of protein were adjusted to pH 5.5 and applied to an FcRn Affinity Column Gen2 (Roche, Basel, Switzerland) using an NGC™ chromatography system (Bio-Rad, Hercules, CA, USA) at a flow rate of 0.5 ml/min. The column was washed with 10 column volumes of equilibration buffer A (20 mM MES, 150 mM NaCl, pH 5.5). Fc-containing proteins bound to the column were eluted using a pH gradient from buffer A to buffer B (20 mM Tris-HCl, 150 mM NaCl, pH 8.8) over 30 column volumes. This chromatography process simulated physiological conditions, with binding at acidic pH (5.5–6.0), resembling the endosomal environment, and release at pH 7.4, mimicking the bloodstream. For complete elution of modified antibodies, the gradient was extended to pH 8.8 [4]. All experiments were conducted at room temperature, and the elution profile was monitored by continuously measuring UV absorbance at 280 nm. Retention time was

defined as the time from sample injection to the maximum detector response of the analyte peak.

ELISA assays

To carry out the ELISA assays, a flat-bottom high-binding 96-well microplate was coated with 50 µl of HER2 at a concentration of 4 µg/ml, diluted in 0.05 M Na_2CO_3 (pH 9.6), and incubated at 4°C for 16 hours. The plate was then blocked with 4% skim milk in PBS at pH 7.4 and incubated at room temperature for one hour. Next, the plate was washed four times with 180 µl of 0.05% PBST (1× PBS and 0.05% Tween20). For analysis of hFcγRs (hFcγRI-GST, hFcγRIIb-GST, hFcγRIIa-131H-GST, hFcγRIIa-131R-GST, hFcγRIIIa-158V-GST, hFcγRIIIa-158F-GST) [5, 6] and hC1q (Quidel, San Diego, CA, USA), 0.05% PBST (pH 7.4) was used, while for analysis of hFcRn at neutral and weakly acidic pH, 0.05% PBST at pH 7.4 and pH 6.0 were used, respectively. Then, 50 µl of trastuzumab-Fc variants, prepared in 1% skim milk in 1× PBS (pH 7.4) or 1× PBS (pH 6.0), were added and incubated at room temperature for one hour. Following this, the plates were washed four times, and 50 µl of serially diluted dimeric hFcγRs, hFcRn-GST or hC1q in 1% skim milk in 1× PBS (pH 7.4) or 1% skim milk in 1× PBS (pH 6.0) were added and incubated at room temperature for one hour. Subsequently, 50 µl of HRP-conjugated antibodies, either goat anti-GST-HRP conjugate (1:5,000) for detecting hFcγRs-GST and hFcRn-GST or sheep anti-hC1q-HRP (1:400) for detecting hC1q, were added. The plates were then washed again, and 50 µl of 1-Step Ultra TMB-ELISA substrate solution was added, followed by the addition of 50 µl

of 2 M H_2SO_4. Absorbance was measured at 450 nm using an Epoch microplate spectrophotometer (BioTek, Winooski, VT, USA).

Analysis of apparent thermostability and in silico immunogenicity

To assess apparent thermostability, a 200× SYPRO Orange working solution was prepared by diluting 5000× original stock dissolved in DMSO in 1× PBS. A total of 5 µl of 200× SYPRO Orange working solution was added to 45 µl of 5 µM of trastuzumab, trastuzumab-Fc variants, or 1× PBS (blank control). The mixtures were thoroughly mixed and transferred into a 0.2 ml semi-skirted 96-well PCR plate/white (Thermo Fisher Scientific, Waltham, MA, USA). All samples were prepared in triplicates. The plate was sealed using optically clear adhesive films (Thermo Fisher Scientific) and centrifuged for 10 sec at 500 × g. Fluorescence signals were recorded using a QuantStudio™ 3 (Thermo Fisher Scientific) while the temperature was increased from 25.0°C to 99.9°C at a rate of 0.03°C/sec. In silico immunogenicity prediction for the wild-type Fc and Fc variants (DHS, PFc29, YML, EML) was conducted using the Immune Epitope Database (IEDB) following a previously described method [2].

Supplementary Figures

Fig. S1 Chromatograms illustrating pH-gradient elution profiles of trastuzumab and trastuzumab-Fc variants. Proteins were eluted from an FcRn affinity column using a pH gradient ranging from 5.5 to 8.8. The retention times were as follows: trastuzumab, 40.64 min (pH 7.65); trastuzumab-EML, 48.00 min (pH 7.97); trastuzumab-PFc29 (pH 8.05), 50.18 min; trastuzumab-YML, 51.24 min (pH 8.09).

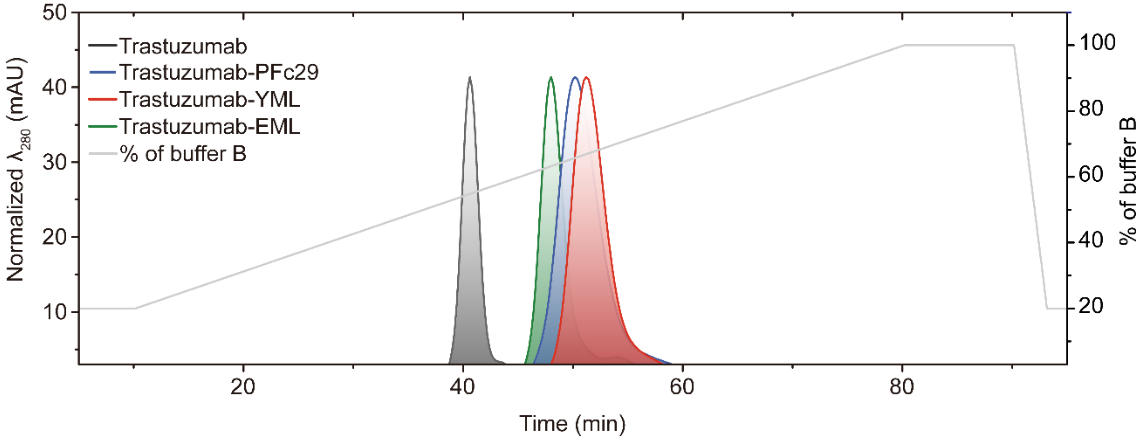

Sample name	Retention time	pH
Trastuzumab	40.64 min	7.65
Trastuzumab-PFc29	50.18 min	8.05
Trastuzumab-YML	51.24 min	8.09
Trastuzumab-EML	48.00 min	7.97

Fig. S2 SPR sensorgrams of hFcRn binding to trastuzumab and trastuzumab-Fc variants under pH 6.0 conditions. a – e Sensorgrams for trastuzumab **a**, trastuzumab-DHS **b**, trastuzumab-PFc29 **c**, trastuzumab-YML **d**, and trastuzumab-EML **e**. Measurements were performed using a Biacore T200 instrument with Series S CM5 sensor chips. Approximately 1,000 RU of each trastuzumab-Fc variant was immobilized, and hFcRn-His, serially diluted from 1,000 to 62.5 nM in 1× PBST (pH 6.0), was injected at a flow rate of 30 µl/min.

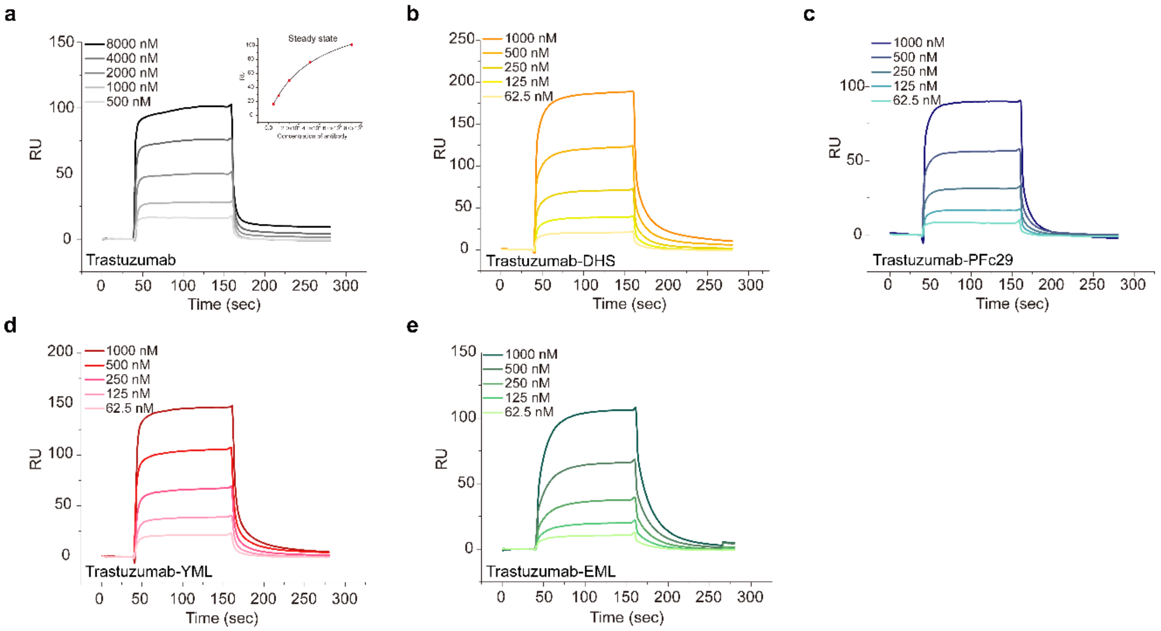

Fig. S3 SEC-HPLC chromatograms of trastuzumab and trastuzumab-Fc variants analyzed using a Biosuit High-Resolution SEC column (7.5 × 300 mm, 250 Å particle size). a – e Chromatograms for trastuzumab **a**, trastuzumab-DHS **b**, trastuzumab-PFc29 **c**, trastuzumab-YML **d**, and trastuzumab-EML **e**.

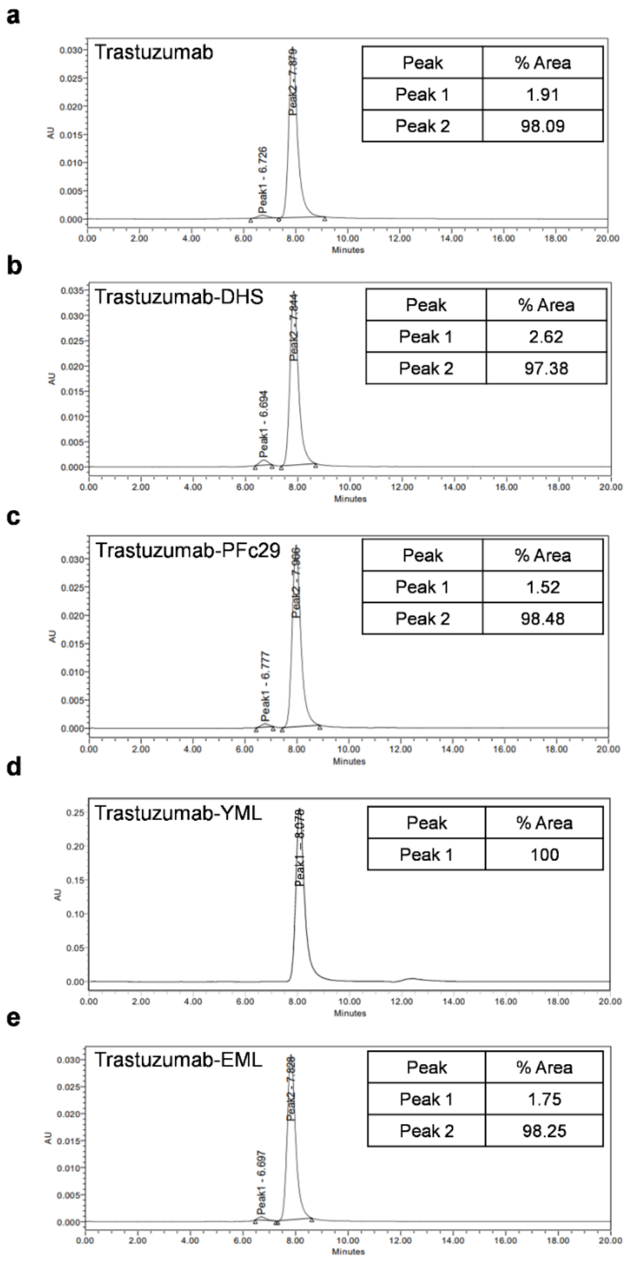

Fig. S4 *N*-linked glycan profile analysis of trastuzumab and trastuzumab-Fc variants. a – **e** LC-MS/MS spectra for trastuzumab **a**, trastuzumab-DHS **b**, trastuzumab-PFc29 **c**, trastuzumab-YML **d**, and trastuzumab-EML **e**.

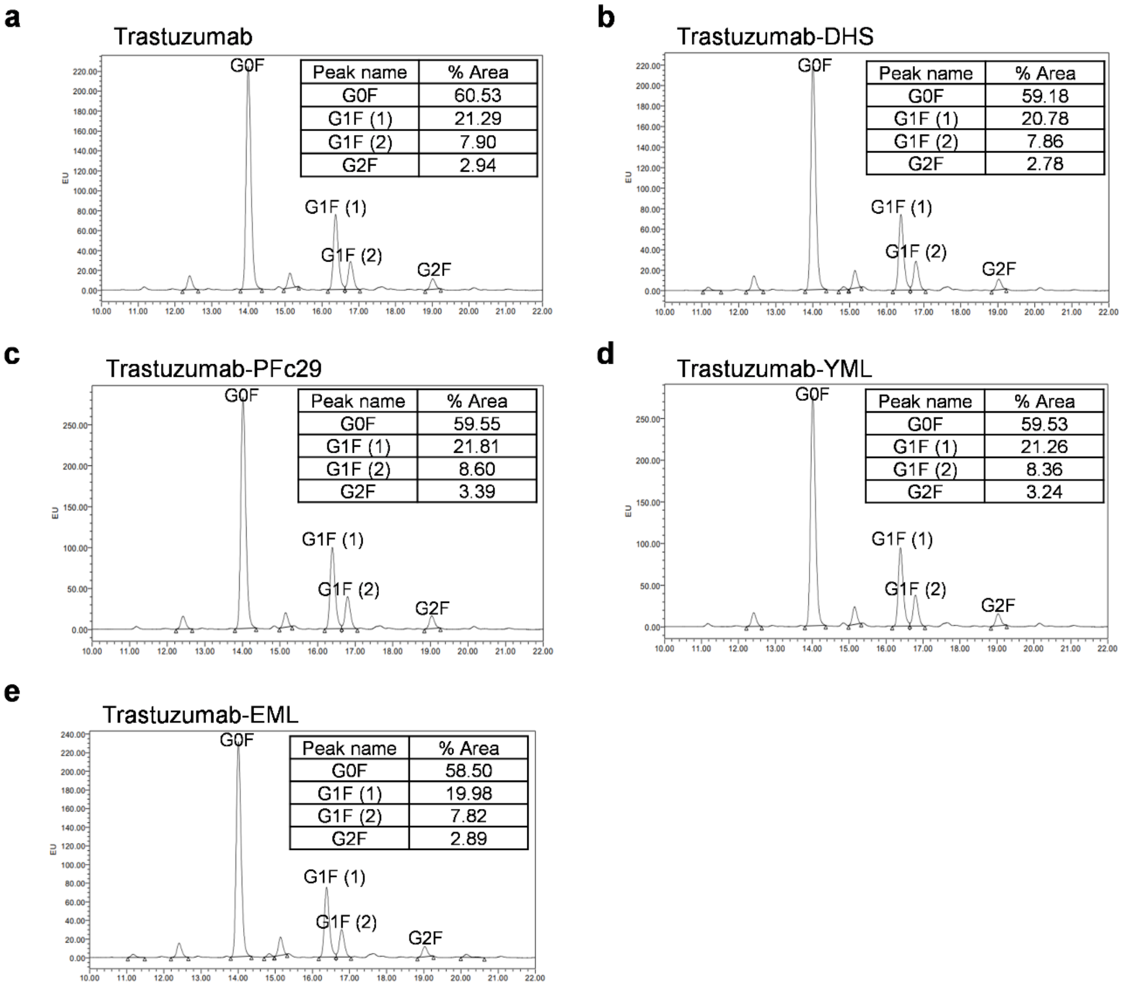

Fig. S5 Thermofluor-based apparent melting temperatures (T_m) of trastuzumab and trastuzumab-Fc variants, including trastuzumab-DHS, trastuzumab-PFc29, trastuzumab-YML, and trastuzumab-EML.

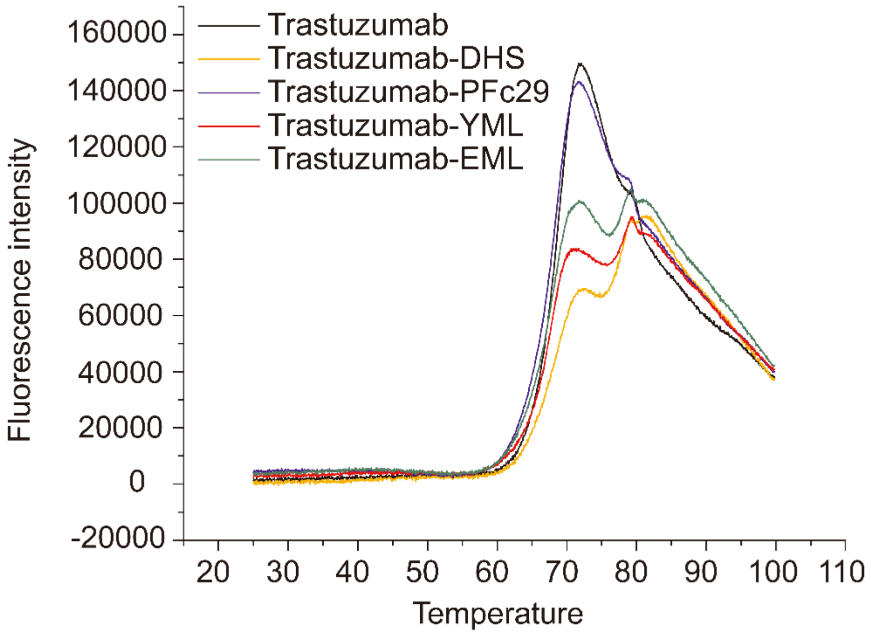

Fig. S6 In silico immunogenicity analysis of Fc variants. a – e Heatmaps for wild-type Fc **a**, DHS **b**, PFc29 **c**, YML **d**, and EML **e**. The x-axis represents the sequence of the 15-mer peptides, while the y-axis denotes the 27 most frequently detected HLA types. Each score is presented as the percentile rank of the predicted binding affinity to MHC class II HLA types [2].

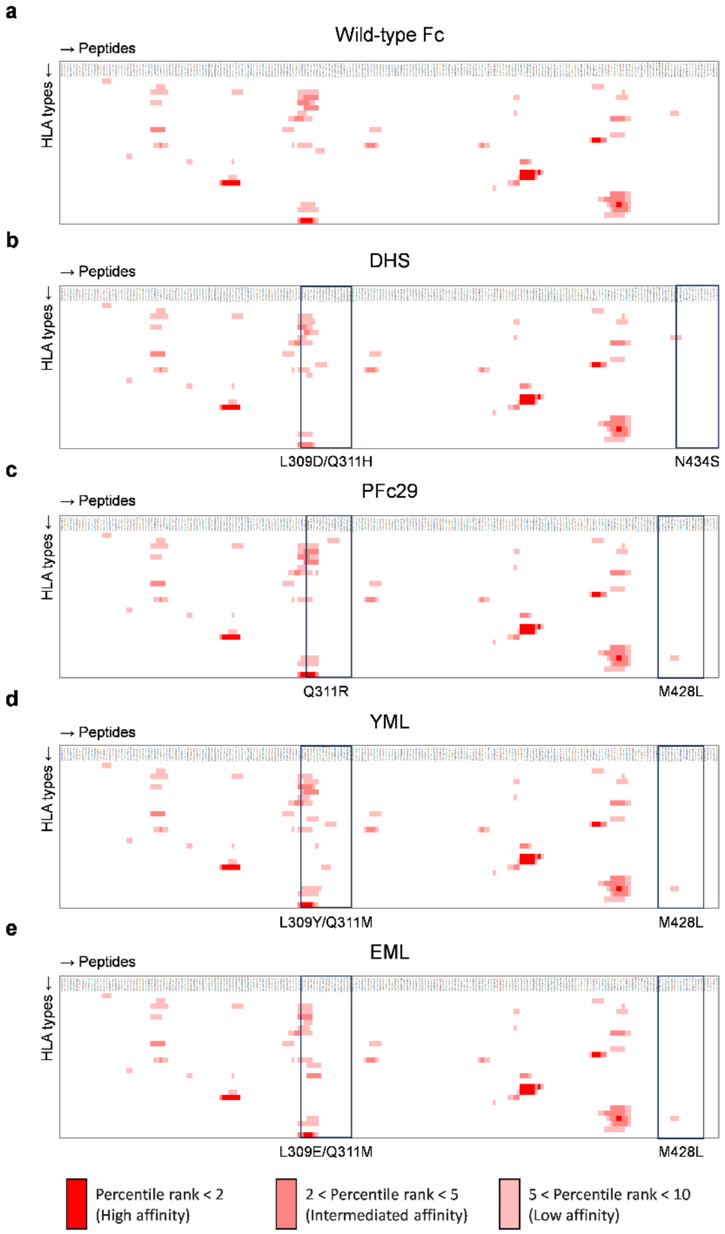

Fig. S7 ELISA assays for Fc ligands binding of trastuzumab and trastuzumab-Fc variants (trastuzumab-DHS, trastuzumab-PFc29, trastuzumab-YML, and trastuzumab-EML) a – g Binding profiles of trastuzumab and trastuzumab-Fc variants to hFcγRI **a**, hFcγRIIa-131H **b**, hFcγRIIa-131R **c**, hFcγRIIb **d**, hFcγRIIIa-158V **e**, hFcγRIIIa-158F **f**, and hC1q **g**. Error bars indicate standard deviations calculated from duplicate samples.

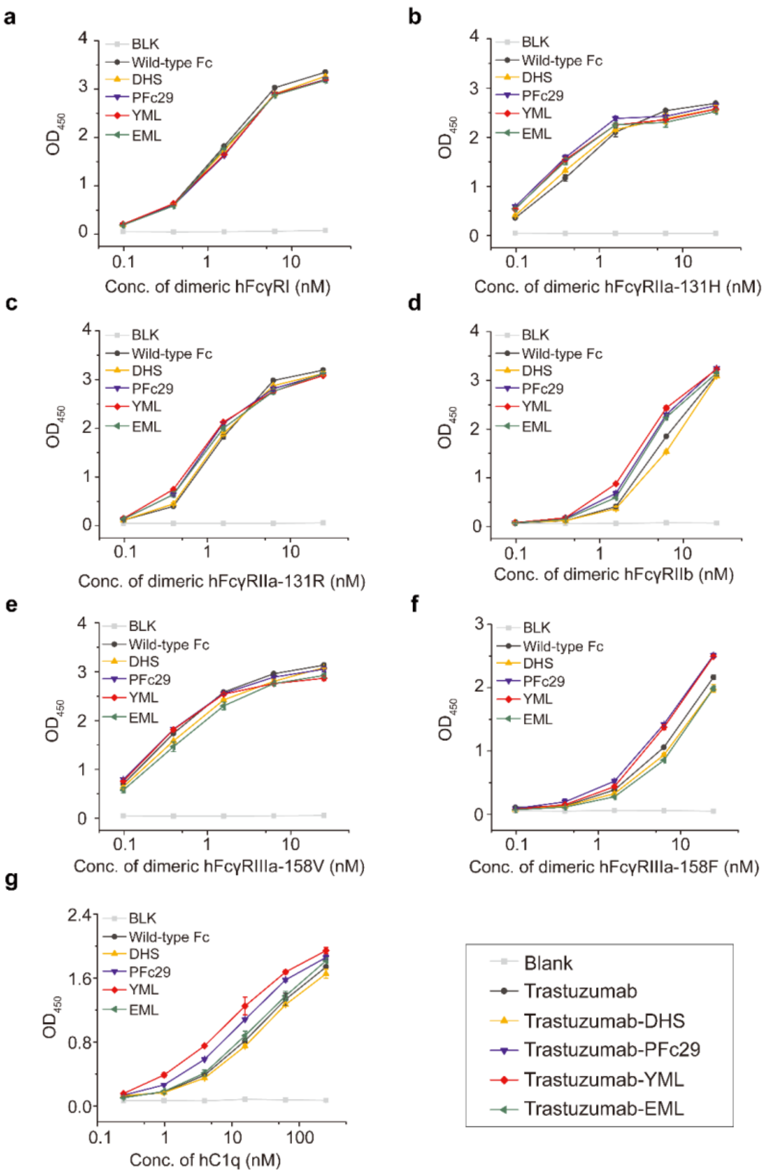

Fig. S8 SEC-HPLC analysis of rituximab and its Fc-engineered variants. a – e Chromatograms of rituximab **a**, rituximab-DHS **b**, rituximab-PFc29 **c**, rituximab-YML **d**, and rituximab-EML **e** analyzed using a Biosuit High-Resolution SEC column.

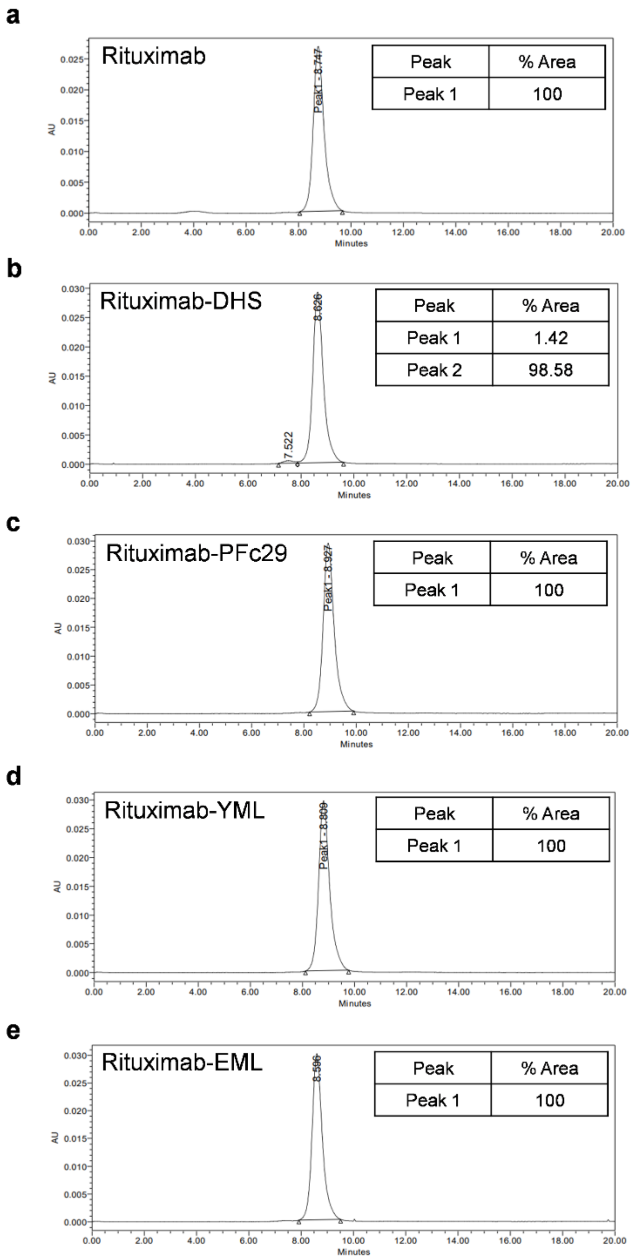

Fig. S9 *N*-linked glycan profiling of rituximab and its Fc-engineered variants. a – e LC-MS/MS spectra of rituximab **a**, rituximab-DHS **b**, rituximab-PFc29 **c**, rituximab-YML **d**, and rituximab-EML **e**.

a

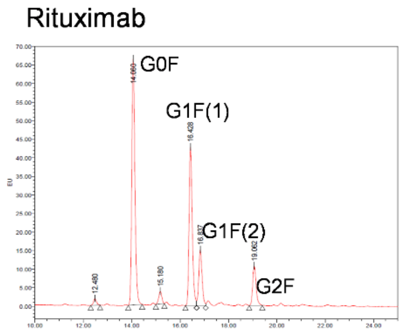

b

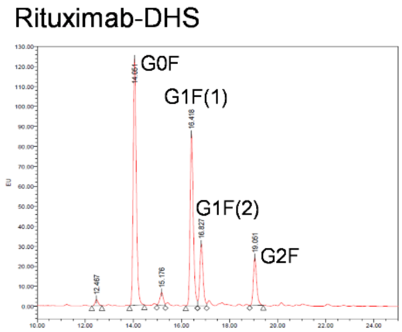

c

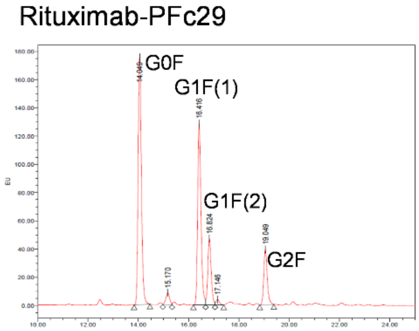

d

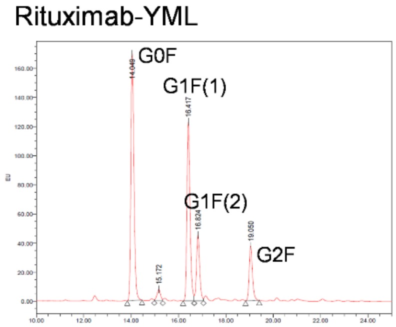

e

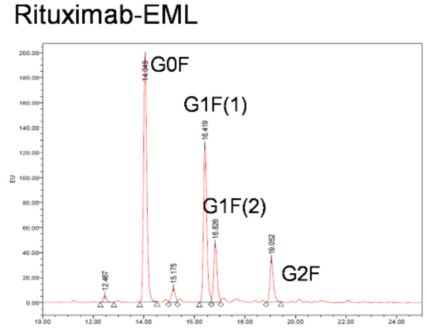

f

	G0F	G1F(1)	G1F(2)	G2F	Area(%)
Rituximab	46.49	30.87	11.01	7.97	96.34
Rituximab-DHS	44.07	31.48	11.55	9.14	96.24
Rituximab-PFc29	42.57	32.09	11.85	10.06	96.57
Rituximab-YML	43.34	32.19	12.04	10.11	97.68
Rituximab-EML	46.04	30.03	11.3	8.48	95.85

Supplementary Tables

Table S1. Plasmids used in this study.

Plasmid name	Relevant characteristics	Reference
pMAZ-hFcRn α chain-His	*Human FcRn α chain-His* gene in pMAZ-IgL	[2]
pMAZ-hβ2m	*Human beta 2 microglobulin* gene in pMAZ-IgL	[2]
pcDNA3-hFcRn-GST	Amp^r CMV promoter, human *FcRn α chain-GST and human beta 2 macroglobulin* gene in pcDNA3	[7]
pMAZ-hFcγRIIa-131H-GST	*Human FcγRIIa*$_{131H}$*-GST* gene in pMAZ-IgL	[5]
pMAZ-hFcγRIIa-131R-GST	*Human FcγRIIa*$_{131R}$*-GST* gene in pMAZ-IgL	[5]
pMAZ-hFcγRIIIa-158V -GST	*Human FcγRIIIa*$_{158V}$*-GST* gene in pMAZ-IgL	[5, 6]
pMAZh-FcγRIIIa-158F-GST	*Human FcγRIIIa*$_{158F}$*-GST* gene in pMAZ-IgL	[5, 6]
pMAZ-hFcγRIIb -GST	*Human FcγRIIb-GST* gene in pMAZ-IgL	[5]
pMAZ-IgL-GlycoT	*Trastuzumab L chain* gene in pMAZ-IgL	[1]
pMAZ-IgH-GlycoT	*Trastuzumab H chain* gene in pMAZ-IgL	[1]
pMAZ-IgH-trastuzumab-PFc29	*Trastuzumab PFc29 mutant H chain* gene in pMAZ-IgL	[2]
pMAZ-IgH-trastuzumab-DHS	*Trastuzumab DHS mutant H chain* gene in pMAZ-IgL	Current study
pMAZ-IgH-trastuzumab-ML	*Trastuzumab ML mutant H chain* gene in pMAZ-IgL	Current study
pMAZ-IgH-trastuzumab-YML	*Trastuzumab YML mutant H chain* gene in pMAZ-IgL	Current study
pMAZ-IgH-trastuzumab-EML	*Trastuzumab EML mutant H chain* gene in pMAZ-IgL	Current study
pMAZ-HER-2-His	*HER-2-His* gene in pMAZ-IgL	Current study
pMAZ-IgL-rituximab	*Rituximab L chain* gene in pMAZ-IgL	[2]
pMAZ-IgH-rituximab	*Rituximab H chain* gene in pMAZ-IgL	[2]
pMAZ-IgH-rituximab-PFc29	*Rituximab PFc29 mutant H chain* gene in pMAZ-IgL	[2]
pMAZ-IgH-rituximab-DHS	*Rituximab DHS mutant H chain* gene in pMAZ-IgL	Current study
pMAZ-IgH-rituximab-YML	*Rituximab YML mutant H chain* gene in pMAZ-IgL	Current study
pMAZ-IgH-rituximab-EML	*Rituximab EML mutant H chain* gene in pMAZ-IgL	Current study

Table S2. Oligonucleotide primers used in this study.

Primer name	Oligonucleotide sequence (5’→3’)
SHK#51	GCTGTATCATCCTCTTCTTGGTAGCAAC
SHK#52	GTGACCGACGGTGAGGACGC
SHK#53	GCGTCCTCACCGTCGGTCACATGGACTGGCTGAATGGCAAGGAGTAC
SHK#54	CAATTTCCTCATTTTATTAGGAAAGGACAGTGGG
SHK#55	GTGGTCAGCGTCCTCACCGTC
SHK#56	GGGCCCTCTAGATCATTTACCCGGGGACAGGGAGAGGCT
SHK#57	GCTGTATCATCCTCTTCTTGGTAGCAAC
SHK#58	CAATTTCCTCATTTTATTAGGAAAGGACAGTGGG
SHK#59	CCACAGGCGCGCACTCCCAGGTCCAGCTCCAACAGCC
SHK#60	GACGGTGAGGACGCTGACCAC
SHK#61	GTGGTCAGCGTCCTCACCGTCTACCACATGGACTGGCTGAATGGC
SHK#62	GTGGTCAGCGTCCTCACCGTCGAGCACATGGACTGGCTGAATGGC

Table S3. On- and off-rate (ΔR/sec) at initial states of association and dissociation.

		Trastuzumab		Trastuzumab-DHS		Trastuzumab-PFc29		Trastuzumab-YML		Trastuzumab-EML	
		ΔR/sec	R^2	ΔR/sec	R^2	ΔR/sec	R^2	ΔR/sec	R^2	ΔR/sec	R^2
Association	Test-1	0.0394	0.9983	0.0722	0.9979	0.0801	0.9977	0.0872	0.9978	0.062	0.9977
	Test-2	0.0427	0.9981	0.0772	0.9977	0.0816	0.9977	0.09	0.9977	0.0626	0.9977
	Test-3	0.037	0.9985	0.0693	0.9981	0.0746	0.998	0.081	0.9981	0.0542	0.9981
	Test-4	0.0464	0.9979	0.08	0.9976	0.0835	0.9975	0.0887	0.9978	0.0642	0.9976
Dissociation	Test-1	-0.0384	0.9996	-0.0716	0.9995	-0.0635	0.9996	-0.0687	0.9997	-0.0623	0.9994
	Test-2	-0.0423	0.9995	-0.0772	0.9994	-0.0664	0.9996	-0.0714	0.9996	-0.0629	0.9994
	Test-3	-0.0358	0.9996	-0.0685	0.9995	-0.0627	0.9996	-0.0682	0.9996	-0.0534	0.9995
	Test-4	-0.0467	0.9994	-0.0803	0.9994	-0.0661	0.9996	-0.0731	0.9996	-0.0647	0.9994

References

1. Jung ST, Reddy ST, Kang TH, Borrok MJ, Sandlie I, Tucker PW, et al. Aglycosylated IgG variants expressed in bacteria that selectively bind FcγRI potentiate tumor cell killing by monocyte-dendritic cells. Proc Natl Acad Sci USA. 2010;107(2):604-9.

2. Ko S, Park S, Sohn MH, Jo M, Ko BJ, Na JH, et al. An Fc variant with two mutations confers prolonged serum half-life and enhanced effector functions on IgG antibodies. Exp Mol Med. 2022;54(11):1850-61.

3. Kawarasaki Y, Griswold KE, Stevenson JD, Selzer T, Benkovic SJ, Iverson BL, et al. Enhanced crossover SCRATCHY: construction and high-throughput screening of a combinatorial library containing multiple non-homologous crossovers. Nucleic Acids Res. 2003;31(21):e126.

4. Cymer F, Schlothauer T, Knaupp A, Beck H. Evaluation of an FcRn affinity chromatographic method for IgG1-type antibodies and evaluation of IgG variants. Bioanalysis. 2017;9(17):1305-17.

5. Jo M, Kwon HS, Lee KH, Lee JC, Jung ST. Engineered aglycosylated full-length IgG Fc variants exhibiting improved FcγRIIIa binding and tumor cell clearance. MAbs. 2018;10(2):278-89.

6. Jung ST, Kelton W, Kang TH, Ng DT, Andersen JT, Sandlie I, et al. Effective phagocytosis of low Her2 tumor cell lines with engineered, aglycosylated IgG displaying high FcγRIIa affinity and selectivity. ACS Chem Biol. 2013;8(2):368-75.

7. Berntzen G, Lunde E, Flobakk M, Andersen JT, Lauvrak V, Sandlie I. Prolonged and increased expression of soluble Fc receptors, IgG and a TCR-Ig fusion protein by transiently transfected adherent 293E cells. J Immunol methods. 2005;298(1-2):93-104.
